# Integrating Radiogenomics and Machine Learning in Musculoskeletal Oncology Care

**DOI:** 10.3390/diagnostics15111377

**Published:** 2025-05-29

**Authors:** Rahul Kumar, Kyle Sporn, Akshay Khanna, Phani Paladugu, Chirag Gowda, Alex Ngo, Ram Jagadeesan, Nasif Zaman, Alireza Tavakkoli

**Affiliations:** 1 Department of Biochemistry and Molecular Biology, University of Miami Miller School of Medicine, Miami, FL 33136, USA; gowdachirag24@gmail.com (C.G.); axn668@med.miami.edu (A.N.); 2 Norton College of Medicine, Upstate Medical University, Syracuse, NY 13210, USA; spornk@upstate.edu; 3 Sidney Kimmel Medical College, Thomas Jefferson University, Philadelphia, PA 19107, USA; aya156@students.jefferson.edu (A.K.); phani.paladugu@students.jefferson.edu (P.P.); 4 Brigham and Women’s Hospital, Boston, MA 02115, USA; 5 Whiting School of Engineering, Johns Hopkins University, Baltimore, MD 21218, USA; ramjagad@cisco.com; 6 Cisco AI Systems, Cisco Inc., San Jose, CA 95134, USA; 7 Department of Computer Science, University of Nevada Reno, Reno, NV 89557, USA; zaman@nevada.unr.edu (N.Z.); tavakkol@unr.edu (A.T.)

**Keywords:** bone cancer diagnosis, AI in medical imaging, radiology and genetics, machine learning in cancer detection, MRI and CT for tumors

## Abstract

Musculoskeletal tumors present a diagnostic challenge due to their rarity, histological diversity, and overlapping imaging features. Accurate characterization is essential for effective treatment planning and prognosis, yet current diagnostic workflows rely heavily on invasive biopsy and subjective radiologic interpretation. This review explores the evolving role of radiogenomics and machine learning in improving diagnostic accuracy for bone and soft tissue tumors. We examine integrating quantitative imaging features from MRI, CT, and PET with genomic and transcriptomic data to enable non-invasive tumor profiling. AI-powered platforms employing convolutional neural networks (CNNs) and radiomic texture analysis show promising results in tumor grading, subtype differentiation (e.g., Osteosarcoma vs. Ewing sarcoma), and predicting mutation signatures (e.g., TP53, RB1). Moreover, we highlight the use of liquid biopsy and circulating tumor DNA (ctDNA) as emerging diagnostic biomarkers, coupled with point-of-care molecular assays, to enable early and accurate detection in low-resource settings. The review concludes by discussing translational barriers, including data harmonization, regulatory challenges, and the need for multi-institutional datasets to validate AI-based diagnostic frameworks. This article synthesizes current advancements and provides a forward-looking view of precision diagnostics in musculoskeletal oncology.

## 1. Introduction

Musculoskeletal tumors present a diagnostic challenge due to their clinical rarity, histological diversity, and overlapping imaging features [1]. This diagnostic complexity often necessitates invasive biopsy and relies heavily on radiologists’ subjective interpretation of imaging studies, which can delay treatment and increase the risk of diagnostic error [2,3] (Table 1). As precision medicine advances, there is growing interest in supplementing conventional diagnostic approaches with emerging technologies that offer greater objectivity, reproducibility, and insight into tumor biology.

This review explores the evolving role of radiogenomics, a field integrating quantitative imaging features with molecular and genomic data, that has emerged as a promising tool to enhance the non-invasive characterization of bone and soft tissue tumors [4]. When combined with machine learning techniques, particularly convolutional neural networks (CNNs) and radiomic texture analysis, these approaches can facilitate more accurate tumor grading, subtype differentiation (e.g., osteosarcoma vs. Ewing sarcoma), and prediction of molecular alterations such as TP53 and RB1 mutations [5,6,7,8,9,10].

Simultaneously, the development of liquid biopsy techniques and the detection of circulating tumor DNA (ctDNA) offer additional avenues for early, minimally invasive tumor profiling. Combined with point-of-care molecular assays, these approaches hold significant potential for improving diagnostic accuracy in high-resource and low-resource settings [11,12,13]. This can improve their widespread adoption and healthcare equity, enabling underfunded facilities to use these technologies to improve patient outcomes and quality of life.

This review synthesizes current advances at the intersection of radiogenomics, artificial intelligence, and molecular diagnostics in musculoskeletal oncology. We also examine translational challenges—such as data standardization, regulatory constraints, and the need for multi-institutional datasets—that must be addressed to integrate these tools into routine clinical practice [14]. By framing these innovations within the context of clinical application, we aim to provide a comprehensive, forward-looking perspective on the future of precision diagnostics for musculoskeletal tumors [15].

## 2. Historical Context and Evolution of Radiogenomics in Oncology

**Figure 1:**
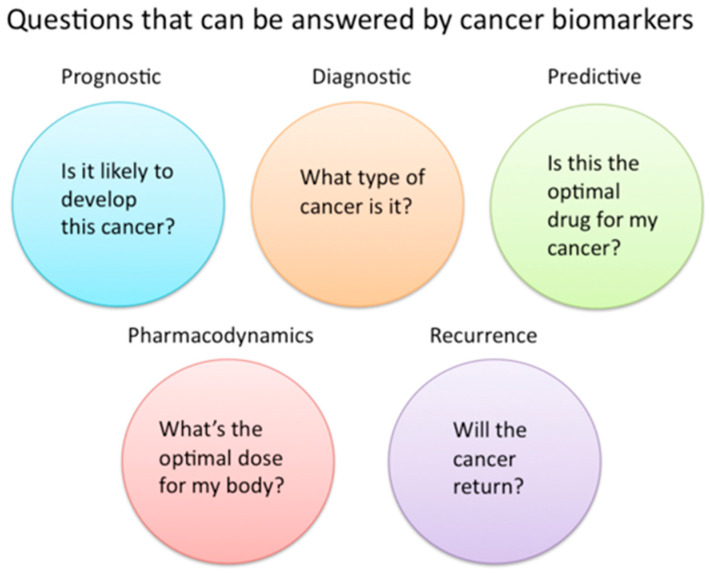
A diagram of the questions that cancer biomarkers can answer. Permission is granted to copy, distribute, and/or modify this document under the terms of the GNU Free Documentation License, Version 1.2 or any later version published by the Free Software Foundation; with no Invariant Sections, no Front-Cover Texts, and no Back-Cover Texts. A copy of the license is included in the section entitled GNU Free Documentation License. This file is licensed under the Creative Commons Attribution-Share Alike 3.0 Unported license with permission from Wikimedia Commons [32].

Radiogenomics serves as a bridge between various scientific disciplines, utilizing non-invasive proxies to reveal underlying genomic and transcriptome patterns [16]. This is achieved using imaging characteristics readily visible on modalities such as MRI, CT, or PET [17,18]. Radiogenomics links macroscopic phenotypes with molecular changes seen on imaging, reducing dependence on invasive tissue biopsies and creating biomarkers for diagnosis, prognosis, and treatment personalization [19,20]. Radiogenomics and radiomics vary conceptually because the former focuses on linking quantitative imaging to genetic data [21]. Radiogenomics uses chromosomal correlations to use these features in a physiological context, while radiomics focuses on feature extraction from images [22]. Studies on prostate cancer by Bourbonne et al. demonstrate how this combination helps to understand tumor biology more than each method alone [23,24]. From early brain tumor applications to cancers of the breast, kidney, colon, and musculoskeletal system, the area has progressed from correlative research to more mechanistically informed approaches [25,26,27]. These developments are based on the notion that imaging technologies can detect phenotypic transformations caused by molecular changes, such as vascularity, cellularity, and metabolic activity [28]. Combining transcriptomics, proteomics, and epigenetics into radiogenomic pipelines has increased their use in uncovering disease pathways and generating personalized treatment, particularly in diagnostically complex cancers (Figure 1) [29,30,31,32].

## 3. Technical Evolution and Methodological Advances

Recent developments in the field involve advances in both imaging and molecular profiling technologies [33]. Early radiogenomic studies were constrained by limited imaging fidelity and basic genetic analyses focused on single-nucleotide variants or restricted gene panels [34]. However, next-generation sequencing and functional imaging modalities have enabled techniques such as diffusion-weighted imaging and MR spectroscopy, which now allow for more nuanced assessments of tumor biology [35,36,37]. Concurrently, clinicians have benefited from advanced computational tools that enable automated feature extraction and have also benefited from novel high-dimensional ML models [38]. Reproducible, algorithm-driven operations mining vast-scale image data for form, texture, and intensity measurements have replaced traditional hand techniques, which tend to have inter-observer variability [39,40].

**Figure 2:**
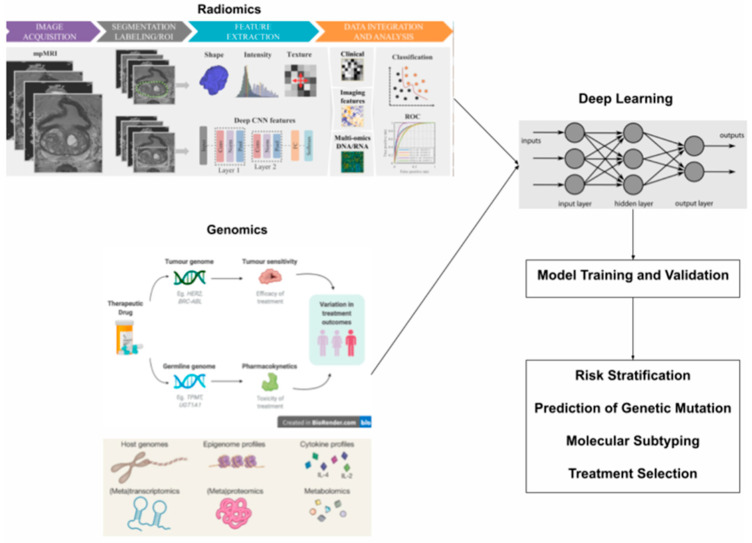
A schematic illustration shows how radiomics is integrated with clinical, genomic, and multi-omics data to build highly accurate predictive models. The diagram outlines a typical radiogenomic workflow, beginning with the collection of clinical, imaging, and genomic datasets. These datasets are standardized and then analyzed collectively to characterize radiomic features and identify distinct molecular associations. These files are licensed under the Creative Commons Attribution 4.0 International license (**top left**) Creative Commons Attribution-Share Alike 4.0 International license (**middle left**), Creative Commons Attribution 4.0 International license (**bottom left**), and the Creative Commons Attribution-Share Alike 3.0 Unported license (**top right**), with permission from Wikimedia Commons [50,51,52,53,54].

Deep learning and convolutional neural networks have enabled end-to-end integration of imaging and genomics, such that predictive features can be taken directly from raw scans [41,42,43]. Integration frameworks have also matured—from isolated correlative models to multi-view learning and kernel fusion techniques that model complex interdependencies between data types [44,45]. This progress is supported by improved data standards and cross-institutional platforms, facilitating larger and more diverse datasets [46]. Newer approaches, including graph neural networks, transfer learning across tumor types, and explainable AI, continue to refine clinical applicability (Figure 2) [47,48,49,50,51,52,53,54].

## 4. Current Applications in Musculoskeletal Cancer Diagnosis and Risk Stratification

Radiogenomics has also begun to influence cancer diagnosis and risk stratification across multiple tumor types [55]. For diagnostic purposes, radiogenomic tools can non-invasively predict the presence of key genetic mutations [56]. For example, KRAS mutations in colorectal cancer correspond with higher FDG absorption on PET imaging and unique MRI morphologic characteristics [57,58]. When biopsy is not possible, KRAS mutations in rectal tumors have been linked to extended axial dimensions and changed shape metrics on pretreatment imaging, guiding appropriate treatment options [59].

For oncologists, early radiogenomic applications show promise in predicting chemotherapy response and disease progression in osteosarcoma [60,61]. Ideally, ML models can generate patient-specific predictive reports [62]. This would be significantly helpful for sharing patient health profiles with other clinicians. Since many tumors undergo metastasis, such as to the brain, breast, and other structures, such models could help identify critical molecular drivers like IDH mutations or HER2 [63,64,65,66]. These tools are particularly valuable in the neoadjuvant setting, where early feedback on treatment efficacy can allow clinicians to adapt strategies before irreversible interventions are made [67].

## 5. Unique Diagnostic Challenges for Bony Tumors

Musculoskeletal tumors pose significant diagnostic challenges due to their histological diversity and anatomical complexity [68]. With only a few thousand new bone and soft tissue sarcoma cases annually in the U.S., most physicians lack extensive exposure to these malignancies, leading to delayed or inaccurate diagnoses [69,70]. Further, due to sarcoma heterogeneity, with over 100 recognized subtypes, each with distinct histopathological and molecular features, high-quality tissue sampling is required but may not always be available, particularly for patients receiving care at community health centers [71,72]. Sarcomas may also arise in bone, cartilage, or soft tissue, and assessing their boundaries, vascular invasion, and resectability can be technically challenging, especially with conventional imaging modalities [73]. Differentiating between tumor types or grades on imaging alone remains unreliable, and standard radiographic criteria may not capture the biological response to therapy [74].

Furthermore, traditional imaging endpoints as defined by the Response Evaluation Criteria in Solid Tumors (RECIST) often fail to reflect true treatment response in sarcomas, particularly following chemotherapy, whereby tumors may become necrotic without shrinking [75]. Artificial intelligence offers promising solutions to these limitations. AI models trained in imaging pattern recognition have enhanced tumor classification, although current methods still require invasive and resource-intensive testing [76,77]. Nevertheless, AI has demonstrated superior specificity compared to clinicians in detecting bone malignancies (86% vs. 64% in internal validations). In comparison, whole-slide image analysis has reduced diagnostic errors in soft tissue tumors by 20–30% compared to assessments by pathologists alone. Additionally, AI-enhanced dynamic MRI can predict chemotherapy response in osteosarcoma with 89% accuracy, aiding surgical planning. These tools show the greatest impact when integrated into multidisciplinary workflows that combine radiological, histopathological, and molecular data (Table 2) [78,79,80,81,82,83].

## 6. Radiomic Feature Extraction

Radiomics transforms conventional imaging into high-dimensional, mineable data by extracting quantitative features using MRI, CT, PET, among other image modalities [84]. The radiomics pipeline—comprising image acquisition, region-of-interest (ROI) segmentation, feature extraction, feature selection, and model construction—requires rigorous clinician standardization at each stage to ensure reproducibility and clinical utility [85,86]. Standardized image acquisition has been foundational, especially as AI/ML models are trained to recognize subtle imaging patterns [87].

Key acquisition parameters must be harmonized to ensure cross-institutional consistency. In MRI, repetition time (TR) and echo time (TE) determine tissue contrast and signal weighting; for instance, a long TR and TE emphasize T2-weighted contrast, which helps visualize edema or necrosis in musculoskeletal tumors [88]. In CT, the reconstruction kernel affects image sharpness and noise, influencing the quality of extracted textural features. The field strength (e.g., 1.5T vs. 3T) in MRI also impacts signal-to-noise ratio and image resolution—factors that influence feature reproducibility [88].

Segmentation is often the most technically demanding component. This is especially true for musculoskeletal tumors, which can span multiple tissue types (bone, cartilage, soft tissue) and exhibit peritumoral edema, necrosis, or infiltrative margins that obscure clear boundaries [89]. While manual segmentation remains the reference standard for accuracy, it is labor-intensive and prone to inter-observer variability. Automated algorithms, including U-net and nnU-net models, offer easily scalable solutions, but can still underperform if given ill-defined or poorly imaged lesions [90,91,92].

Once the ROI is established, clinicians can extract radiomic features. These include the following:

- First-order features such as mean intensity, skewness, and kurtosis are derived from histogram analysis of voxel intensity distributions. For example, kurtosis quantifies the peakedness of intensity distributions, where higher values may indicate regions of dense cellularity. A tumor with high intensity kurtosis may exhibit regions of dense cellularity. PyRadiomics provides the formula for kurtosis as $\frac{\mu^{4}}{\sigma^{4}}$ where $\mu^{4}$ is the fourth central moment. Diffusion kurtosis imaging studies demonstrate that kurtosis metrics strongly correlate with glioma cellularity and proliferation indices [93,94].
- Shape descriptors such as sphericity (Equation (1)) and elongation (Equation (2)) quantify tumor geometry. These metrics correlate with invasiveness, as compact/spherical tumors often exhibit less aggressive behavior compared to irregular or elongated masses [95,96].
  (1) $$\mathrm{Sphericity}=\frac{Average\;radial\;length}{Standard\;Deviation\;Radial\;Length}$$

Equation (1): Equation for Tumor Sphericity [95].

(2) $$\mathrm{Eccentricity}=\frac{Longest\;Axis}{Orthogonal\;Axis}$$

Equation (2): Equation for Tumor Eccentricity [95].

- Second-order and higher-order texture features are derived from the gray-level co-occurrence matrix (GLCM) and gray-level run-length matrix (GLRLM). GLCM features include contrast (highlighting local intensity variation, with higher values in rough/textured regions), entropy (indicating randomness in intensity distributions, where higher values indicate heterogeneity), and homogeneity (measuring uniformity, with lower values found in heterogeneous tumors) [86]. GLRLM features quantify runs of similar intensity levels, such as short-run emphasis (highlights fine textures, reflecting necrosis of fibrosis) [86] or long-run low gray-level emphasis (captures extended regions of low intensity, associated with cystic components or edema) [86,97].

**Figure 3:**
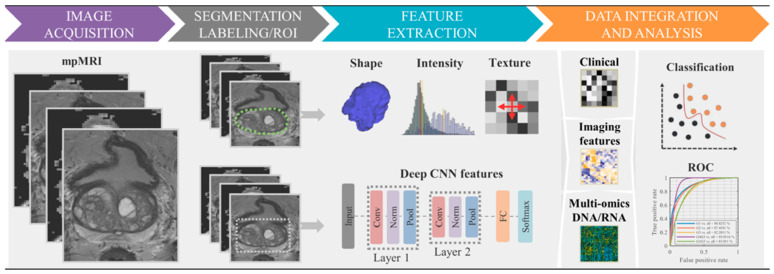
A flowchart of the standard radiomics model. (1) Multiparametric MRI (mpMRI) image acquisition. (2) Segmentation: tumor labeling—green/white contour. (3) Imaging features extraction using shape, texture, and/or deep features derived from convolutional neural network layers. (4) Clinical, radiomic features, molecular data for statistical analyses, based on significance test and classifier models, to identify relevant features for predicting the clinical outcome (e.g., Gleason score). This file is licensed under the Creative Commons Attribution 4.0 International license with permission from Wikimedia Commons [50].

These textural features are sensitive to microstructural changes in tumors, such as necrosis (heterogeneous regions with high entropy) or fibrosis (organized patterns with low contrast) [86]. For example, EGFR-mutant lung adenocarcinomas show higher heterogeneity through more GLCM entropy and short-run emphasis [86,97]. These features help create a non-invasive phenotypic fingerprint of the tumor, often correlated with histopathologic findings like grade, mitotic rate, or vascular invasion [98,99,100]. When aggregated, they enrich traditional radiologic interpretation and serve as valuable inputs for ML models to augment traditional radiologic evaluation. They serve as a good substrate for future predictive modeling aiming to predict outcomes or therapeutic responses (Figure 3) [50,101].

## 7. Advanced Imaging Modalities for Feature Extraction

MRI remains the gold standard in imaging soft tissue tumors due to its superior tissue resolution and versatility [102]. Standard sequences like T1- and T2-weighted sequences and gadolinium-based contrast-enhanced imaging form the imaging backbone, offering detailed anatomical and perfusion information [103]. Functional MRI techniques such as diffusion-weighted imaging (DWI) and apparent diffusion coefficient (ADC) mapping extend the imaging arsenal and can provide oncologists with quantitative assessments of cellularity and membrane integrity, with low ADC values often indicating that high tumor cellularity has shown strong correlations with tumor grade and chemotherapy response in sarcomas [104,105].

On the other hand, CT imaging is less sensitive for soft tissue contrast but excels in evaluating osseous structures and mineralization patterns [106]. Its standardized intensity scale, measured in Hounsfield units (Equation (3)), offers clinicians a reproducible density-based feature extraction report [107].

(3) $$\mathrm{Hounsfield}\;\mathrm{unit}\;\mathrm{formula}=HU=\left(\frac{\mu_{material}-\mu_{water}}{\mu_{water}}\right)*1000$$

Equation (3): Hounsfield Unit Formula [108].

A radiologist can subsequently analyze patterns of cortical destruction, mineralization, and periosteal reaction in the bony tumor, which can provide diagnostic and staging insights [109]. Recent developments in radiology, such as dual-energy CT and spectral CT imaging, add another dimension by differentiating materials (e.g., calcium and iodine). Perfusion CT offers insights into vascularity, though its applications remain limited as physicians must balance excessive radiation dose with quantity of information [110,111].

PET imaging, especially in hybrid PET/CT or PET/MRI formats, adds a critical metabolic layer to the radiomics framework [112]. Fluorodeoxyglucose-PET (FDG-PET) assesses metabolic activity, with elevated uptake often indicating higher-grade, metabolically active, aggressive sarcomas [112,113]. Radiomics features such as metabolic tumor volume (MTV), total lesion glycolysis (TLG) (Equation (4)), and heterogeneity indices offer valuable information on uneven tracer uptake, reflecting tumor aggressiveness and patient prognosis [114,115]. Metabolic tumor volume, introduced by Larson et al. [116], is the metabolically active volume of the tumor segmented through FDG-PET scans and is useful for predicting patient outcome and assessing treatment responses. Two methods have been introduced to measure MTV: fixed relative and background. The fixed relative method is simpler and observer-independent but underestimates the volume of heterogeneous tumors and overestimates low signal-to-noise lesions. This method is effective for delineating tumor volume in idealized spherical tumors with homogenous tracer uptake.

On the other hand, the background method offers the option to adjust the scan threshold, but it is more time-consuming and has relatively low reproducibility. This method is useful for real-world cases [116,117]. The growing use of alternative PET tracers targeting proliferation, hypoxia, or amino acid metabolism (e.g., 18F-FMISO or 11C-Methionine) further extends the biological specificity of PET-based radiomics [118].

(4) $$\mathrm{Total}\;\mathrm{Lesion}\;\mathrm{Glycolysis}={SUV}_{mean}*MTV$$

Equation (4): Equation for Total Lesion Glycolysis [119].

**Figure 4:**
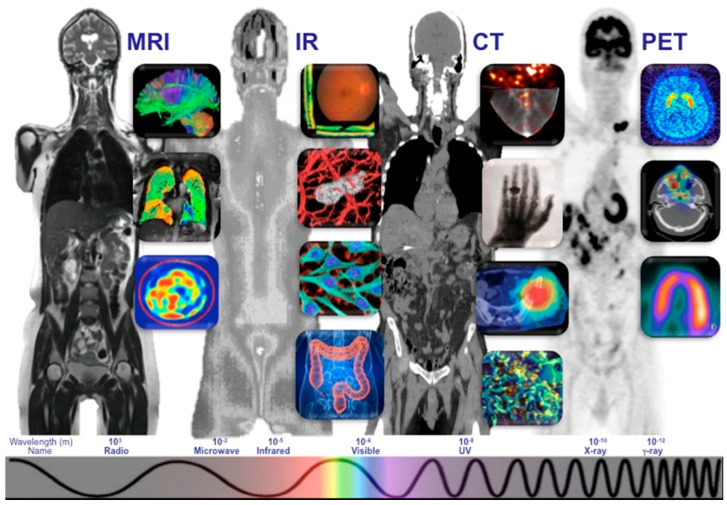
The montage, originally featured on the *Journal of Medical Imaging*’s cover, showcases diverse imaging techniques—from MRI, CT, PET, and SPECT to photoacoustic imaging, optical microscopy, and electron microscopy. Each contributes unique insights into the human body, aided by advances in algorithms, machine learning, and data fusion. This file is licensed under the Creative Commons Attribution 4.0 International license with permission from Wikimedia Commons.

Integrating PET features with those derived from anatomical (CT, MRI), functional (DWI, ADC), and metabolic (PET) imaging enables a multiparametric view of tumor biology (Figure 1) [120]. Combining DWI, dynamic contrast-enhanced MRI (DCE-MRI), and MR spectroscopy, for instance, enriches radiomics analysis by capturing diverse aspects of tumor physiology like cellularity, perfusion, and metabolic shifts [121]. The challenge, however, lies in integrating these multimodal datasets cohesively [122]. Data fusion strategies, such as early fusion (raw image integration), feature-level fusion (combining extracted features), and decision-level fusion (aggregating model outputs), are being actively explored to optimize model performance and clinical applicability (Figure 4) [123,124].

## 8. Quantitative Feature Analysis and Standardization

While innovative radiomic techniques have helped extract high-dimensional quantitative features from medical imaging, this same dimensionality introduces the risk of model overfitting, especially in small datasets [125]. Therefore, careful feature selection enhances predictive model generalizability and interpretability [126]. Some statistical approaches that clinicians use to isolate predictive features include filter-based relevance ranking, wrapper-based optimization, and embedded regularization techniques like least absolute shrinkage and selection operator (LASSO) regression for regularization [127,128]. When properly selected, radiomic signatures derived from T1-weighted, T2-weighted, and contrast-enhanced MRI sequences have demonstrated diagnostic accuracies comparable to those of radiologists in distinguishing between benign and malignant soft tissue lesions [129].

However, fine-grained classification tasks (e.g., identifying sarcoma subtypes) remain constrained by limited cohort sizes and the heterogeneity of available imaging datasets across institutions [130]. One source of variability is the differences in scanner hardware, acquisition parameters, reconstruction protocols, and image reconstruction techniques [131]. To address these issues, several batch effect correction methods like ComBat have been repurposed to normalize feature distributions across image sources [132]. More sophisticated algorithms such as NestedComBat and GMM-ComBat refine these adjustments by modeling nonlinear, multimodal feature distributions [133].

**Figure 5:**
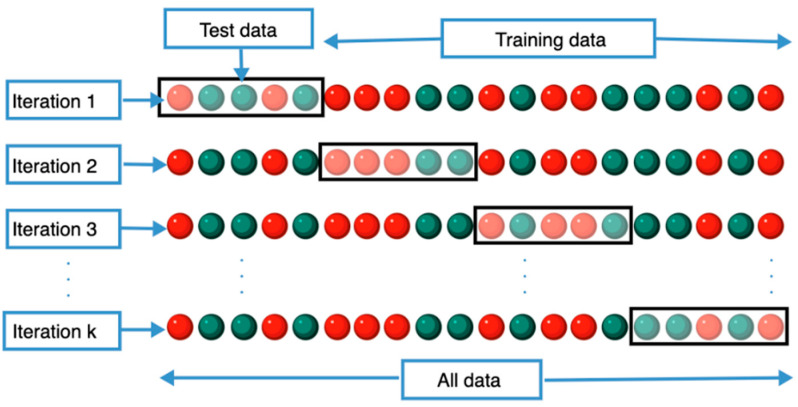
K-fold cross-validation method. This file is licensed under the Creative Commons Attribution-Share Alike 4.0 International license with permission from Wikimedia Commons [139].

Another important consideration is the reproducibility of features under different segmentation scenarios. Many tumors infiltrate dense tissues, making consistent region-of-interest (ROI) definition a challenge [134]. Studies have shown that first-order histogram features and second-order texture descriptors (e.g., GLCM, GLRLM) demonstrate higher reproducibility than shape-based and higher-order wavelet features [97,135]. Robustness is often quantified using test–retest protocols, multi-reader segmentations, and phantom studies to ensure fidelity across imaging sessions [136]. Given these challenges, comprehensive radiomics validation frameworks must integrate internal validation (e.g., k-fold cross-validation) (Figure 5), external validation on independent datasets, and eventual clinical validation through prospective trials [137,138].

Ultimately, the translational utility of radiomics models extends beyond traditional performance metrics such as AUC, F1-score, and model calibration and measurable impact on clinical decision-making [139]. While early studies show promising diagnostic equivalency with expert radiologists, reliable subtype-level prediction and embedding these tools into therapeutic workflows require further refinement [140]. Improving the reproducibility and standardization of the entire radiomics pipelines will be key to realizing their full potential in tumor characterization and precision workflows [50,141].

## 9. Standardized Workflow and Quality Control

Building on the need for reproducibility and standardization outlined above, implementing rigorous workflow and quality control procedures is vital for research and clinical deployment. In large academic centers, quality control assessments can ensure that acquired images have sufficient signal-to-noise ratio, minimal artifacts, and consistent timing relative to biopsies or treatment initiation [142].

Segmentation, a critical step in feature extraction, also demands consistency. Inter-reader reliability is necessary when using manual or semi-automated segmentation approaches to ensure reproducibility across different operators [143]. Additionally, automated segmentation algorithms should be benchmarked against expert-labeled datasets to ensure fidelity [144].

**Figure 6:**
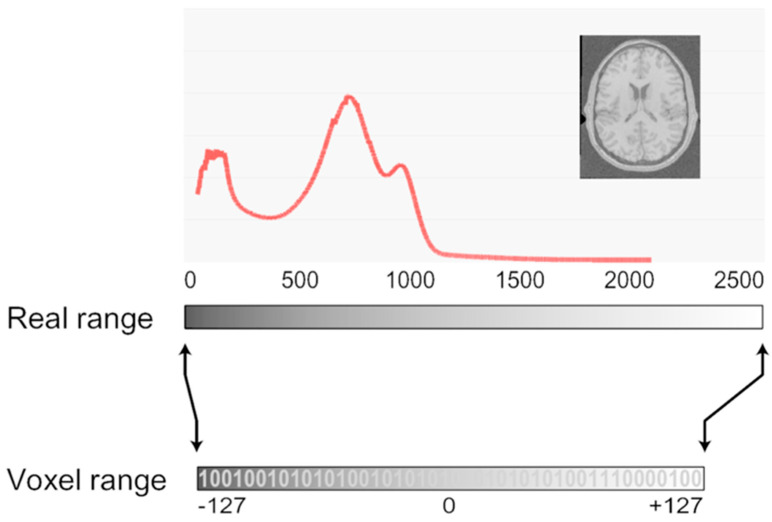
Mapping from voxel to real data range. This file is licensed under the Creative Commons Attribution-Share Alike 2.5 Generic license under permission from Wikimedia Commons [146].

In the pre-processing phase of feature extraction, standardizing steps such as voxel resampling, intensity normalization, and discretization is crucial, as these can substantially influence the computed features (Figure 6) [145,146].

Following established guidelines, especially from the Image Biomarker Standardization Initiative (IBSI), helps ensure consistency and comparability across studies [147]. Additionally, open-source platforms like PyRadiomics and CapTK can provide validated, reproducible tools that radiologists can use to compute features against, enabling broader accessibility and consistency in implementation [148].

During model development, sound statistical rationale must inform feature selection and dimensionality reduction to avoid overfitting and enhance interpretability [149]. Cross-validation and dimensionality reduction techniques are indispensable, especially in limited datasets. Final, real-world evaluation using independent, multi-institutional test sets provides critical insight into a model’s generalizability and clinical applicability [150,151]. These quality control measures are the backbone of a reliable radiomics pipeline and are integral to realizing the promise of precision imaging.

## 10. Genomic and Transcriptomic Integration with Imaging Features

Musculoskeletal tumors are a biologically heterogeneous group of neoplasms defined by diverse genetic, epigenetic, and immunologic mechanisms that reflect distinct cellular origins and differentiation trajectories [152]. They are broadly classified into two molecular categories: genomically simple tumors driven by specific genetic alterations, such as the pathognomonic EWSR1-FLI1 translocation seen in Ewing sarcoma, and those with complex karyotypes typified by chromosomal instability, as in osteosarcoma and undifferentiated pleomorphic sarcoma [153,154]. In the former, fusion oncoproteins function as aberrant transcription factors, disrupting normal gene regulatory networks that govern proliferation and differentiation, whereas in the latter, chaotic genomic rearrangements (including chromothripsis) and recurrent inactivation of tumor suppressors like TP53 and RB1 promote genomic instability and therapeutic resistance [155,156]. High-throughput sequencing in osteosarcoma has revealed frequent perturbations in canonical signaling pathways such as WNT/β-catenin, PI3K/AKT/mTOR, and Notch, which collectively shape transcriptomic landscapes influencing angiogenesis, immune evasion, and metastatic behavior [157,158].

Epigenetic dysregulation further complicates the molecular architecture of sarcomas; fusion proteins like EWSR1-FLI1 and SS18-SSX co-opt chromatin remodeling complexes and distort enhancer topologies, leading to widespread transcriptional reprogramming [159]. Insights from the ENCODE Project have underscored the oncogenic roles of non-coding regulatory elements and disrupted 3D chromatin conformations in many tumors [160]. Compounding this complexity, sarcomas exhibit heterogeneous immune microenvironments, with variable immune cell infiltration and checkpoint molecule expression influencing immunotherapy responsiveness [161]. Cytokine profiling has identified inflammatory mediators such as IL-1Ra, IL-6, IL-8, and TNF-α as prognostic indicators in osteosarcoma, with distinct cytokine endotypes correlating with metastatic risk and survival outcomes [162,163,164,165,166]. Collectively, these molecular, epigenomic, and immunologic insights are reshaping the paradigm of sarcoma biology and illuminating new targets for biomarker-guided, multimodal therapeutic strategies [167].

Emerging technologies are refining our understanding of this complexity. Spatial transcriptomics using 18,000-gene panels enables 5 µm resolution mapping of tumor-immune interactions in bone lesions, while radiomic features such as entropy and texture correlate with tumor mutational burden and neoantigen load. Additionally, when integrated with MRI, methylation profiling reduces diagnostic ambiguity in undifferentiated sarcomas by up to 40%. Collectively, these molecular, epigenomic, and immunologic insights are reshaping the paradigm of sarcoma biology and informing the development of biomarker-guided, multimodal therapeutic strategies (Table 3) [167,168,169,170,171,172,173,174,175].

## 11. Correlation of Imaging Phenotypes with Genomic Signatures

Advanced radiogenomic models increasingly incorporate deep learning techniques, including but not limited to CNNs, to enhance the detection of subtle imaging patterns tied to genomic features [176]. While this has been demonstrated most prominently in gliomas—where CNNs have accurately predicted IDH mutations, MGMT methylation, and 1p/19q codeletion—the same principles are being extended to musculoskeletal tumors [177,178].

Transcriptomic profiling offers a seemingly beneficial avenue for linking imaging features with tumor biology by revealing the active gene expression landscape [179]. Unlike genomic sequencing, which catalogs DNA alterations, transcriptomics highlights the functional output of the genome, enabling clinicians to track pathways active in tumor growth, immune modulation, and treatment response [180]. In sarcomas, transcriptomic signatures have been used to stratify patients by histologic subtype, grade, and prognosis [181]. These patterns often reflect deregulated processes such as hypoxia, inflammation, and cell cycle dysregulation—also visible on imaging through signal intensity patterns, enhancement profiles, or diffusion metrics [182].

Recent studies integrating transcriptomics with radiomics in soft tissue sarcomas have uncovered radiomic clusters associated with aggressive phenotypes, even when not directly linked to specific gene expression profiles [183]. For example, groups defined by deep radiomic features often overlap with transcriptomic signatures characterized by the upregulation of pro-tumorigenic pathways [184]. In osteosarcoma, specific MRI features correlate with the expression of angiogenic and metastatic genes [185]. These relationships may be rooted in molecular mechanisms such as transcriptional reprogramming by fusion proteins, enhancer hijacking, or chromatin remodeling—all of which influence tumor phenotype and can be reflected in imaging data [186].

Imaging phenotypes such as margin irregularity, necrosis, and signal intensity on MRI correlate strongly with genomic signatures indicative of tumor aggressiveness and prognosis. Radiogenomic integration thus enables non-invasive prediction of molecular subtypes and survival outcomes, offering a powerful alternative to biopsy, which may be limited by sampling error or accessibility. While much of this work has been modeled in breast cancer, the translational potential for musculoskeletal tumors is substantial, given overlapping imaging and molecular characteristics. Furthermore, combining liquid biopsy markers—such as circulating tumor DNA (ctDNA) and circulating tumor cells (CTCs)—with imaging modalities enhances disease monitoring, particularly for tracking bone metastases (Table 4) [172,187,188,189].

Alternative splicing, RNA editing, and post-transcriptional regulation introduce additional layers of transcriptomic complexity that may modulate imaging phenotypes [190]. Importantly, the same genomic locus can produce multiple protein isoforms with varying biological effects, potentially altering texture or diffusion [191]. These dynamic molecular processes align with ENCODE’s expanded definition of genes as sets of overlapping functional products rather than singular units [192].

## 12. Inflammatory Cytokine Profiles and Their Predictive Value

Cytokines and chemokines secreted by tumor and stromal cells orchestrate immune responses, modulate angiogenesis, and contribute to metastatic behavior [193]. In osteosarcomas, elevated levels of cytokines IL-6 and TNF-α have been linked to more aggressive disease, including metastasis and reduced survival [164,165,166,194,195]. These markers may reflect underlying biological processes, such as activation of the NF-κB or JAK/STAT pathways, that drive tumor progression [196]. IL-6’s role in promoting angiogenesis and immune evasion suggests that high serum levels may indicate a more hostile tumor phenotype [197]. This suggests that tumor-immune dysregulation is not merely a response to tumor presence, but an intrinsic part of cancer biology with significant prognostic implications [198].

Importantly, these immune signatures may also manifest in imaging phenotypes [199]. For example, tumors with high inflammatory activity often show greater contrast enhancement, increased peritumoral edema, and distinct diffusion profiles [200]. These features, quantified through radiomics, could indirectly indicate the underlying cytokine milieu [201]. By combining serum biomarkers with imaging-derived features, researchers are beginning to construct integrative models that more accurately reflect tumor biology [202]. In soft tissue sarcomas, radiomic–transcriptomic clusters associated with inflammation and hypoxia were linked to poor prognosis, suggesting that imaging can capture molecular processes such as immune activation [203].

Further supporting this integrative approach, specific cytokines—including IL-18, IP-10, MCP-1, M-CSF, MIG, SCGFβ, Eotaxin, and IL-7—are consistently elevated in untreated musculoskeletal inflammatory tumors and tend to decrease with treatment, correlating with disease activity and severity. IL-18R1 expression is particularly notable in dermatomyositis, observed in muscle tissue and peripheral blood, suggesting its potential as a predictive biomarker for certain tumor subtypes. Overall, pro-inflammatory cytokines such as TNF-α, IL-6, and IL-1 appear broadly implicated in musculoskeletal tumor progression and may serve as valuable prognostic indicators across multiple sarcoma types [164,165,166,204].

## 13. Machine Learning Algorithms in Musculoskeletal Tumor Classification

ML has become an increasingly valuable tool in oncologic practice, particularly in classifying and prognosticating musculoskeletal tumors through predictive modeling based on labeled datasets. By leveraging labeled datasets that represent tumor type, grade, or molecular alterations, ML models learn complex associations between image-derived features extracted from radiological, genomic, and clinical inputs [204,205]. Two primary categories dominate this field and are highly effective in data-limited settings due to interpretability and performance efficiency: classical ML algorithms and deep learning (DL) models [206,207].

## 14. Classical Machine Learning Algorithms

**Figure 7:**
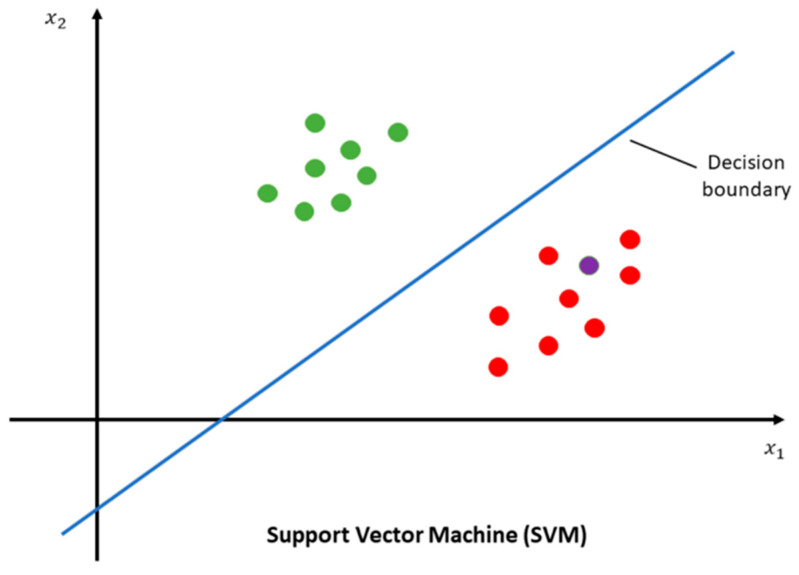
Graph demonstrating support vector machine. This file is licensed under the Creative Commons Attribution-Share Alike 4.0 International license with permission from Wikimedia Commons [210].

**Figure 8:**
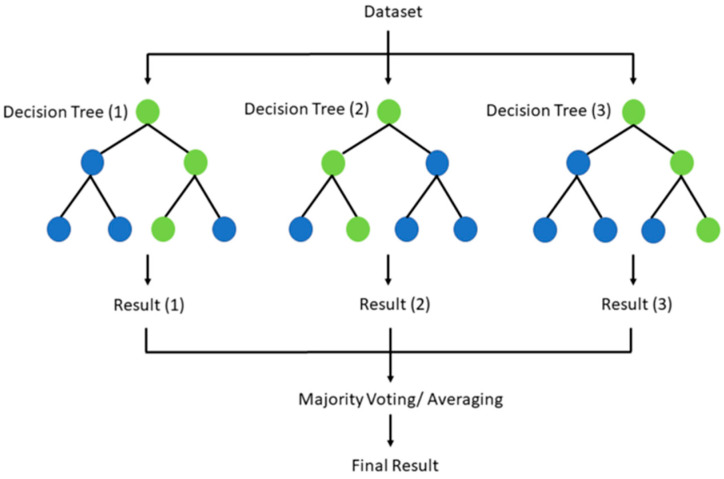
Diagram demonstrating how random forests generate results [210].

Classical ML methods, including support vector machines (SVMs) (Figure 7) [208], random forests (Figure 8) [209], and logistic regression, remain widely used in musculoskeletal classification, especially in limited data availability settings [206,207].

**Figure 9:**
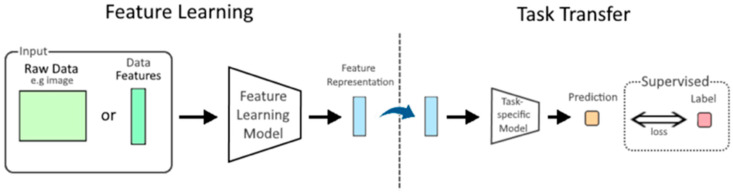
This diagram illustrates the concept of feature learning in machine learning, where models automatically extract informative representations from raw data (e.g., images or text) or pre-processed features. The goal is to improve task-specific performance or training efficiency compared to using unprocessed data directly, similar to the approach used in transfer learning. This file is licensed under the Creative Commons Attribution-Share Alike 4.0 International license with permission from Wikimedia Commons [216].

These models excel due to their relative simplicity, computational efficiency, and interpretability—qualities that are particularly important in clinical environments. They perform well in binary or small-class tasks (e.g., distinguishing benign from malignant lesions) and are less susceptible to overfitting when regularization techniques like LASSO are applied [210,211,212]. Feature selection is critical in radiogenomic processes because of the large dimensionality of input data, with approaches such as LASSO efficiently decreasing feature space while keeping predictive fidelity (Figure 9) [212]. These sparse modeling techniques reduce overfitting and increase translational applicability [213]. Classical models also offer more transparency in decision-making, facilitating clinical trust and regulatory approval. However, their performance may plateau in more complex classification tasks, such as differentiating between sarcoma subtypes or predicting multi-omics-defined risk profiles [211,214]. To establish generalizability, extensive validation techniques—including k-fold cross-validation and external cohort testing—are required, especially when accounting for inter-institutional heterogeneity in imaging protocols [214]. As datasets grow and multimodal integration becomes more common, supervised ML is positioned to underlie future efforts in diagnoses, risk assessment, and decision-making [215].

## 15. Deep Learning Models

In contrast, deep learning models, especially CNNs, have demonstrated diagnostic parity with expert radiologists [210]. Unlike classical models, DL models can autonomously extract hierarchical features directly from raw imaging data without explicit feature engineering. Advanced architectures, including deep radiomics features such as convolutional autoencoder (CAE) and hybrid half-supervised CAE (HSCAE) networks, have shown the ability to identify aggressive phenotypes and predict overall survival in musculoskeletal tumors [217,218]. When combined with transcriptomic or other omics data, these deep features can offer superior prognostic performance compared to single-modality approaches [219,220,221].

**Figure 10:**
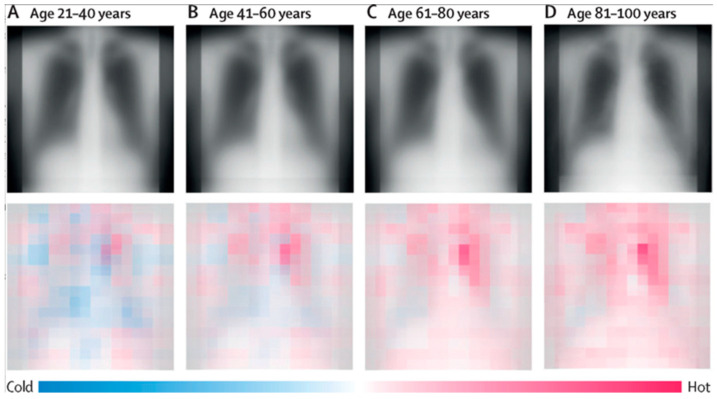
Average saliency maps and chest X-rays from an institution’s external test dataset are shown by 20-year age groups. The top row displays the mean chest radiographs per group, while the bottom row presents the corresponding average saliency maps. Warmer regions in the saliency maps highlight features associated with older age, whereas cooler regions indicate traits linked to younger age. This file is licensed under the Creative Commons Attribution 4.0 International license with permission from Wikimedia Commons [229].

Despite these advantages, deep learning models present unique challenges. They require extensive, well-annotated data to achieve generalizability, which is difficult to obtain in uncommon tumors or retrospective studies [222]. Approaches such as transfer learning, data augmentation, and synthetic image generation using GANs have been employed to overcome these limitations [45,223,224,225]. Another significant concern is model interpretability [226]. Many deep models operate as “black boxes”, preventing transparency in AI clinical decision-making and hindering widespread adoption [226]. To facilitate clinical acceptance, visualization tools such as saliency maps (Figure 10), class activation maps (CAMs), and attention mechanisms are used to highlight image regions critical to model decisions, offering biological insight into predictions [226,227,228,229].

Ultimately, both classical and DL approaches have roles to play. Classical models may be more appropriate for low-dimensional datasets requiring explainable outputs, while DL models—such as CNNs, multitask architectures, and ResNet50—offer greater scalability and adaptability in high-dimensional, multimodal scenarios [178,228]. With continued advances in explainability and data availability, CNNs and other DL models are projected to play an increasingly important role in decision support for musculoskeletal oncology (Table 5) [230].

Indeed, recent studies have demonstrated that DL models can achieve diagnostic accuracy comparable to or exceeding that of experienced musculoskeletal radiologists when classifying tumors on radiographs and MRI. Radiomics-based machine learning has also proven highly effective in distinguishing between tumor subtypes and grades, performing at a level like expert human readers [231]. Automated segmentation tools, such as the Multi-Scale Attention Pyramid Network (MSAPN), enhance workflow efficiency while maintaining high classification accuracy [232,233]. Additionally, integrating standardized reporting systems—such as BTI-RADS 2.0—with machine learning frameworks supports consistent and reproducible grading, further aiding clinical decision-making across diverse healthcare settings (Table 6) [80,231,232,233,234,235].

## 16. Unsupervised Learning for Pattern Discovery

Unsupervised learning offers distinct advantages for discovering novel patterns, especially when labeled data are limited or when exploring heterogeneity within complex datasets [236]. These algorithms identify latent structures in imaging or molecular data without relying on predefined outcomes [237]. Techniques such as k-means clustering, hierarchical clustering, and dimensionality reduction methods like PCA, t-SNE, and UMAP allow researchers to explore high-dimensional data, visualize hidden groupings, and generate new hypotheses [238,239].

Unsupervised techniques have also proven useful: a prospective cytokine profiling study in sarcoma patients used hierarchical clustering to identify immune endotypes with distinct survival outcomes [240]. Patients in one cluster—marked by elevated CXCL5, CXCL12, and MIF—had significantly worse event-free and overall survival, suggesting that inflammatory profiling can uncover clinically relevant subgroups [241]. These findings support using unsupervised methods for discovering immune signatures that may influence treatment response [242].

## 17. Translational Barriers and Technical Challenges

One of the main barriers inhibiting AI’s use in imaging is harmonizing imaging and molecular data across heterogeneous acquisition protocols, as scanner type, sequence parameters, and reconstruction algorithms (e.g., TE/TR, slice thickness, kernel filters) can introduce non-biological variability [243] (Table 3). Fortunately, many statistical harmonization techniques like ComBat and GMM-ComBat have been adapted from genomics to mitigate scanner-induced batch effects in radiomics without degrading prognostic signal [244,245]. However, these require consistent pre-processing pipelines and comprehensive metadata [246].

Molecularly speaking, sample handling, library preparation, and sequencing depth can compromise transcriptomic reproducibility [247]. Additionally, clinicians would require fusion methods to align multimodal, multi-scale data [248]. Early, intermediate, and late fusion models must account for differences in dimensionality (e.g., high-dimensional RNA-seq vs. structured imaging features) and scale (e.g., continuous vs. categorical vs. spatial inputs) [249]. While intermediate-level integration, leveraging autoencoders or canonical correlation analysis, shows promise, robust performance requires careful normalization, missing data imputation, and cross-modal feature selection [250,251].

Model interpretability and segmentation variability also remain non-trivial [252]. CNNs and transformers excel in tumor detection and classification, but function as black boxes [253]. Explainability tools—e.g., Grad-CAM, SHAP, and attention heatmaps—are increasingly deployed to map model saliency to anatomical or radiomic domains [254,255]. Additionally, segmentation, which is a prerequisite for most radiomics workflows, remains a bottleneck [256]. Manual and semi-automated tumor delineation suffers from inter-observer variability, especially for infiltrative tumors; fully automated approaches remain challenged by heterogeneity and imaging artifacts [257]. Standardizing segmentation protocols and integrating neural attention-based contouring (e.g., using nnUNet frameworks) are emerging solutions [258].

Clinical deployment is further limited by ethical and legal concerns [259]. Adaptive artificial intelligence systems via “predetermined change control plans”, currently included in the FDA’s 2019 proposed regulatory framework for modifications to AI/ML-based software as a Medical Device (SaMD) architecture, pose difficulties for auditability and validation given continual learning from real-world data [260]. Good Machine Learning Practice (GMLP) concepts stress data variety, algorithmic fairness, and transparency, but require infrastructure and governance frameworks not yet widespread in clinical imaging environments [261]. Moreover, algorithmic bias—stemming from imbalanced training cohorts or confounding imaging variables—poses risks of inequitable outcomes, necessitating bias-aware modeling and subgroup validation [262].

Privacy-preserving federated learning, scalable low-cost algorithms for resource-limited environments, and integrated diagnostics platforms integrating liquid biopsy data (e.g., ctDNA, CAPP-seq assays) with radiomics should ultimately take the front stage [263,264]. Prospective, multicenter trials are desperately required to determine how the clinical effects of AI-driven tools affect treatment choices and patient outcomes [265]. Multimodal fusion, harmonized datasets, and robust interpretability methods will be foundational in translating musculoskeletal imaging AI from proof-of-concept to routine clinical deployment (Table 7) [266].

## 18. Conclusions

Radiogenomics, AI, and ML progressively enable non-invasive, quantitative assessment of soft tissue malignancies with significant accuracy. Radiomics has become a strong foundation for extracting high-dimensional image features capturing microarchitectural and textural traits beyond human awareness. Together with ML models—from interpretable algorithms like random forests to high-capacity convolutional neural networks—these characteristics assist tumor classification, grading, subtype distinction, and outcome prediction. Deep learning shows diagnostic performance on par with experienced radiologists while preserving scalability for use in high-throughput environments, particularly when used on multimodal inputs like hybrid PET-CT or multiparametric MRI.

Models integrating imaging features with transcriptomic, epigenetic, and mutational data give molecular insights for physically challenging cancers like spinal or pelvic sarcomas, reducing the issues linked with recurring biopsies. This methodology combines advanced regression and classification models, enhanced by dimensionality reduction methods such as LASSO and PCA, and multimodal fusion methodologies enabling virtual molecular profiling. Liquid biopsy analytics increases circulating tumor surveillance capabilities by utilizing either ctDNA sequencing with high-sensitivity CAPP-Seq or translocation detection utilizing long-read nanopore assays. The dynamic interaction of fluid and image-based markers makes real-time monitoring of minimum residual illness, clonal evolution, and treatment response conceivable.

These technologies have numerous useful applications. Imaging-derived models might direct surgical margins, assess chemosensitivity, and choose patients for limb-sparing surgeries versus more aggressive resections. Combining point-of-care molecular diagnostics with AI-augmented ultrasonic or low-field MRI allows fast and reasonably priced triage in community or resource-limited settings. Translation still presents challenges. In imaging systems, segmentation methods, scanner platforms, and uniformity impact reproducibility. Harmonizing methods like ComBat and GMM-ComBat lower batch effects but rely on consistent pretreatment techniques and datasets strong in metadata. Strong normalization and cross-platform calibration are called for when variance in genomic data from differences in sequencing depth, alignment methods, or library preparation calls for significant noise.

In future developments, interpretability, equality, and clinical validation must be front and center. Gain clinician confidence and regulatory approval using explainable artificial intelligence (XAI) methods like SHAP values, attention heatmaps, and class activation maps. Low-resource contexts need lightweight designs (e.g., MobileNet, quantized models) or federated learning to maintain data sovereignty while allowing cross-site training. Prospective, multicenter studies assessing combined AI–radiogenomic systems for clinical decision assistance, risk assessment, and therapy customization are sorely lacking. Sustainable adoption depends critically on rigorous implementation science, regulatory adherence to the FDA’s SaMD advice, and ethical protections addressing algorithmic bias and data privacy.

## Figures and Tables

**Table 1:** The unique diagnostic challenges regarding bony tumors. This table highlights specific clinical and imaging-related obstacles that complicate accurate diagnosis, treatment planning, and access to care. Specific emphasis is placed on imaging limitations, tumor biology, and barriers to AI deployment in real-world settings.

Diagnostic Challenge	Clinical Impact
Low clinical exposure due to rarity (e.g., Ewing sarcoma, chordoma)	Delayed or missed diagnosis due to limited familiarity among general radiologists and clinicians
High histological heterogeneity (e.g., differentiation between liposarcoma subtypes)	Requires expert pathology and imaging interpretation; misclassification can affect treatment selection
Difficult tissue access in underserved or resource-limited settings	Suboptimal biopsy access and diagnostic delays, leading to delayed interventions
Variable anatomical origin (bone, cartilage, soft tissue, e.g., osteosarcoma, synovial sarcoma)	Complicates imaging interpretation, surgical planning, and multidisciplinary coordination
Poor delineation of tumor margins with conventional imaging (e.g., T1-weighted imaging)	Inadequate surgical margin assessment increases the risk of local recurrence or incomplete resection
Unreliable differentiation of tumor grade/type via imaging alone	May lead to incorrect prognostication or therapy selection without histological confirmation
Inaccuracy of Response Evaluation Criteria in Solid Tumors (RECIST) in measuring therapy response, especially in necrotic or cystic tumors	Can lead to misjudgment of therapeutic effectiveness, potentially affecting patient inclusion in clinical trials or treatment continuation
Invasiveness, data requirements, and resource demands of current AI model training (eg., deep learning with 3D MRI)	Limits the scalability and adoption of AI tools in low-resource or community settings

**Table 2:** Diagnostic challenges for diagnosing soft tissue and bony tumors and the AI solution that has recently emerged to mitigate these issues and bring forth earlier treatment options for patients, leading to better prognoses.

Diagnostic Challenge	Tumor Type	AI Solution	References
Morphological overlap with benign lesions	Soft tissue tumors	Deep learning models analyze histopathology slides to distinguish benign vs. malignant subtypes with 84–95% accuracy	[78]
Ambiguous radiographic features	Bone tumors	Convolutional neural networks (CNNs) detect subtle patterns in X-rays/MRI (e.g., “moth-eaten” appearance seen in Ewing sarcoma vs. “sunburst” appearance in Osteosarcoma) with 86–91% specificity	[79,80]
Molecular heterogeneity	Both	Machine learning integrates transcriptomic, immunohistochemical, and imaging data to predict tumor-specific mutations (e.g., EWSR1 translocations in Ewing sarcoma)	[78,81,82]
Inter-observer variability in biopsy interpretation	Both	AI algorithms standardize biopsy analysis by quantifying cellular features (e.g., mitotic count, necrosis) to reduce diagnostic discordance	[79,81]
Time-intensive manual tumor grading	Soft tissue tumors	Automated segmentation tools measure tumor volume and heterogeneity on MRI/CT, reducing grading time by 30–50%	[80,82]
Differentiating round-cell tumors	Bone tumors	AI-driven FISH/RT-PCR prioritization identifies high-risk molecular markers (e.g., CD99 for Ewing sarcoma) with 95% concordance	[79,83]

**Table 3:** Integrating genomic and transcriptomic data with imaging features enhances diagnostic precision, prognostic stratification, and therapeutic targeting in musculoskeletal tumors [176].

Tumor Type	Genomic/Transcriptomic Feature	Imaging Feature	Integration Method	Reference
Osteosarcoma	PLK1 pathway activation; glucose metabolism genes	Heterogeneous Contrast Enhancement on MRI/CT	Multi-omic pathway analysis (WES, RNA-seq, drug screens)	[168]
Soft Tissue Sarcoma	Gene fusions (e.g., EWSR1); methylation profiles	Necrosis/hemorrhage patterns on T2-weighted MRI	Spatial transcriptomics + MRI radiomics	[169,170]
Multiple Myeloma	Copy number alterations; HLA-G/LILRB1 interactions	Focal bone lesions on PET/CT	Spatial multi-omics (IF, IMC, LC/MS proteomics)	[171]
Bone Metastases	Circulating tumor DNA (ctDNA); AR-variant expression	Osteolytic/osteoblastic changes on CT	Radiogenomic correlation (CTCs, cfDNA)	[172,173,174]
Sarcomas (general)	Subtype-specific mutations (e.g., TP53, RB1)	Tumor texture/heterogeneity on dynamic contrast MRI	Multi-omics clustering (PET/MRI + RNA-seq)	[175]

**Table 4:** Table summarizing correlation of imaging phenotypes with genomic signatures in musculoskeletal tumors [172,187,188,189].

Imaging Phenotype	Genomic Signature/Molecular Feature	Tumor Type/Context	Key Findings/Correlation	Reference
Irregular tumor margins and intratumoral necrosis	Poor response-associated gene expression profiles	Various cancers including musculoskeletal tumors	Presence correlates with poor neoadjuvant chemotherapy response; imaging features reflect aggressive genomic behavior	[187]
Tumor heterogeneity on MRI (contrast enhancement patterns)	Gene expression subtypes related to immune response (e.g., interferon-related genes)	Breast cancer (model for musculoskeletal tumors)	Heterogeneous enhancement correlates with specific gene expression subtypes linked to prognosis	[188]
Low or absent T2 signal intensity on MRI	Poor prognosis gene sets including van’t Veer 70-gene signature	Breast cancer (analogous to fibrotic/malignant features in musculoskeletal tumors)	Low T2 signal correlates with poor prognosis gene signatures, reflecting collagen-rich fibrotic tissue	[188]
Radiomic texture and entropy features	Tumor mutational burden (TMB) and neoantigen load	Bone malignancies and sarcomas	Radiomic features correlate with genomic markers of tumor aggressiveness and immune evasion	[172]
Spatial heterogeneity in imaging	Intratumoral genomic subclones identified by transcriptomics	Breast cancer (framework applicable to musculoskeletal tumors)	Radiogenomic signatures link imaging heterogeneity to genomic subclone composition, predicting survival outcomes	[189]
Imaging features of bone metastases (osteolytic/osteoblastic changes)	Circulating tumor DNA (ctDNA) and gene expression alterations	Bone metastases	Imaging phenotypes correlate with liquid biopsy genomic markers, enabling non-invasive monitoring	[172]
Peritumoral edema and necrosis on MRI	Gene expression signatures related to hypoxia and wound healing	Various solid tumors including sarcomas	Imaging features correlate with gene sets associated with tumor microenvironment and aggressive biology	[187,188]

**Table 5:** Radiomic feature extraction. This table presents the sequential steps and imaging technologies essential to the radiomics pipeline used in musculoskeletal tumor evaluation. It highlights critical considerations such as standardizing imaging parameters, addressing segmentation complexity, extracting robust texture and shape features, integrating advanced modalities like PET/MRI, and harmonizing datasets for model reproducibility. Additionally, the table captures the evolving integration of transcriptomic data and immune signatures into radiomic analyses, emphasizing the expanding role of AI and deep learning in transforming image-derived features into clinically actionable insights.

Radiomic Process Step or Modality	Significance for Musculoskeletal Tumor Assessment	Key Considerations/Outputs
Imaging Acquisition Standardization	Minimizes inter-scan and inter-site variability for model training and cross-center reproducibility	Protocol harmonization; consistent voxel spacing, timing, and field strength
ROI Segmentation (Manual, Semi-/Automated)	Defines tumor boundaries for downstream analysis; crucial for accurate feature mapping, prone to variability	Affected by operator variability; accuracy impacts all subsequent model inputs
Quantitative Feature Extraction	Converts image data into quantifiable metrics representing tumor morphology and texture	Includes shape, intensity, texture (GLCM, GLRLM), and wavelet features
MRI Functional Imaging (e.g., DWI and ADC mapping)	Reflects tumor cellularity and treatment response using ADC values and other water diffusion metrics	ADC values inversely correlate with cell density; useful in response monitoring
CT with Dual-Energy and Spectral Imaging	Enhances tissue characterization, particularly in bone tumors and mineral content	Enables separation of materials (e.g., calcium vs. iodine); improves lesion conspicuity
PET/CT and PET/MRI Metabolic Profiling	Evaluates tumors aggressiveness and viability through metabolic markers such as MTV and TLG	Includes metabolic tumor volume (MTV) and total lesion glycolysis (TLG)
Multiparametric Imaging Integration	Provides a comprehensive view by combining functional, anatomical, and metabolic imaging for robust modeling	Fuses MRI, PET, and CT data for superior classification and response assessment
Feature Selection and Overfitting Prevention	Prioritizes robust, predictive features to avoid in small datasets	Uses LASSO, recursive feature elimination, or embedded CNN layers to improve generalizability
Standardization using ComBat and NestedComBat Techniques	Adjusts for scanner and protocol-induced variability to enable pooled analysis	Batch effect correlation enhances multi-site model compatibility
Transcriptomic Integration with Imaging	Links radiomic phenotypes with gene expression for personalized diagnosis based on molecular subtypes	Enables radiogenomic profiling and non-invasive surrogate biomarkers of oncogenic pathways
Immune-Related Radiomic Signatures	Maps imaging biomarkers associated with immune infiltration or systemic inflammation	Correlates with IL-6, TNF-α expression; may predict immunotherapy response
Deep Learning and CNN-Driven Radiogenomics	Enhances automated classification and prognostication from raw scans	Improves classification, survival prediction, and uncovering latent imaging-genomic patterns

**Table 6:** Table summarizing main machine learning algorithms used in musculoskeletal tumor classification, their applications, and performance [172,187,188,189].

Algorithm/Model	Application	Performance	Reference
Deep Convolutional Neural Network (CNN)	Classifying benign vs. malignant bone lesions, subtyping (e.g., cartilaginous vs. osteogenic)	Top 1 error rate of 0.25; 93% accuracy in matrix classification, outperforming average radiologists (70%)	[80]
Pre-trained ResNet50 Classifier	Predicting malignant potential of bone tumors on MRI	93.7% accuracy (T1-weighted); 86.7% accuracy (T2-weighted)	[80]
Ensemble Deep Learning Network	Integrating multicenter radiographs and clinical features	High accuracy for primary bone tumor classification	[234]
Multitask Deep Learning Model	Simultaneous segmentation and classification on radiographs	80.2% accuracy, 62.9% sensitivity, 88.2% specificity; performance comparable to fellowship-trained radiologists	[235]
Radiomics-based Machine Learning	Differentiating atypical cartilaginous neoplasms from chondrosarcoma (MRI/CT)	92% accuracy (MRI), 78% AUC (CT), similar to specialized radiologists (98% accuracy)	[231]
Automated Segmentation (MSAPN) + Radiomics	MRI-based segmentation and classification	Dice score 0.871 (test set); 0.890 accuracy for benign vs. malignant classification	[232]
Risk Stratification System (BTI-RADS 2.0) + ML	Standardized bone lesion grading on CT/MRI	Sensitivity of 96% for malignant lesions; F1-score 0.81 (slightly below radiologists at 0.83)	[233]

**Table 7:** Translational barriers and technical challenges in musculoskeletal imaging AI. This table summarizes critical barriers that impede the clinical translation of AI-enhanced musculoskeletal imaging tools. These challenges span technical inconsistencies in imaging protocols and molecular data quality, methodological hurdles in data fusion and model interpretability, and broader issues related to regulatory frameworks, algorithmic fairness, and deployment in low-resource settings. Addressing these challenges through harmonization, equitable model development, and regulatory reform is crucial for the safe and scalable implementation of AI-driven diagnostics.

Translational Barrier or Challenge	Description	Implication for Clinical Translation
Heterogeneous Imaging Acquisition Protocols	Variations in scanner models, sequences, and reconstruction algorithms across sites	Introduces batch effects and non-biological variability
Inconsistent Transcriptomic Profiling Methods	Differences in RNA extraction, sequencing platforms, and bioinformatic pipelines	Compromises data quality and model reliability
Integrating Multimodal Data (Imaging + Genomics)	Challenges in aligning data with different resolutions, formats, and missing values	Limits comprehensive tumor profiling; demands advanced fusion models and data imputation strategies
Low Model Interpretability and Explainability	DL models often operate as “black boxes” without transparent decision logic	Limits clinician trust and slows regulatory acceptance; explainable AI (XAI) methods are essential (e.g., saliency maps)
Segmentation Variability and Lack of Standardization	Inter- and intra-observe variability in manual or semi-automated tumor delineation	Reduces feature reproducibility; highlights the need for robust auto-segmentation algorithms and consensus protocols
Regulatory Hurdles and Clinical Validation Challenges for Adaptive AI Models	Continuously learning systems evolve post-deployment, making validation and certification complex	Raises legal and ethical concerns; requires new regulatory frameworks and post-market surveillance mechanisms
Algorithmic Bias and Performance Disparities Acros Subgroups	Underrepresentation of demographic subgroups during training	Can lead to inaccurate predictions in minorities; underscores the need for diverse datasets and fairness audits
Lack of Federated Learning Implementation in Low-Resource Clinical Settings	Limited infrastructure in low- and middle-income countries for decentralized model training	Exacerbates global disparities in AI access; federated learning could enable secure, local model deployment

## Data Availability

No new data were created or analyzed in this study. Data sharing is not applicable to this article.

## References

[1] Grimer R.J., Briggs T.W. (2010). Earlier Diagnosis of Bone and Soft-Tissue Tumours. J. Bone Jt. Surg. Br..

[2] Rechl H., Kirchhoff C., Wörtler K., Lenze U., Töpfer A., von Eisenhart-Rothe R. (2011). Diagnosis of Malignant Bone and Soft Tissue Tumors. Orthopäde.

[3] Clark M.A., Thomas J.M. (2005). Delay in Referral to a Specialist Soft-Tissue Sarcoma Unit. Eur. J. Surg. Oncol..

[4] Shui L., Ren H., Yang X., Li J., Chen Z., Yi C., Zhu H., Shui P. (2021). The Era of Radiogenomics in Precision Medicine: An Emerging Approach to Support Diagnosis, Treatment Decisions, and Prognostication in Oncology. Front. Oncol..

[5] Trivizakis E., Papadakis G.Z., Souglakos I., Papanikolaou N., Koumakis L., Spandidos D.A., Tsatsakis A., Karantanas A.H., Marias K. (2020). Artificial Intelligence Radiogenomics for Advancing Precision and Effectiveness in Oncologic Care (Review). Int. J. Oncol..

[6] Ferro M., de Cobelli O., Vartolomei M.D., Lucarelli G., Crocetto F., Barone B., Sciarra A., Del Giudice F., Muto M., Maggi M. (2021). Prostate Cancer Radiogenomics—From Imaging to Molecular Characterization. Int. J. Mol. Sci..

[7] Kickingereder P., Bonekamp D., Nowosielski M., Kratz A., Sill M., Burth S., Wick A., Eidel O., Schlemmer H., Radbruch A. (2016). Radiogenomics of Glioblastoma: Machine Learning-Based Classification of Molecular Characteristics by Using Multiparametric and Multiregional MR Imaging Features. Radiology.

[8] Hinterwimmer F., Guenther M., Consalvo S., Neumann J., Gersing A., Wörtler K., von Eisenhart-Rothe R., Burgkart R., Rueckert D. (2024). Applications of Machine Learning for Imaging-Driven Diagnosis of Musculoskeletal Malignancies—A Scoping Review. BMC Musculoskelet. Disord..

[9] Breden S., Hinterwimmer F., Consalvo S., Neumann J., Knebel C., von Eisenhart-Rothe R., Burgkart R.H., Lenze U. (2023). Deep Learning-Based Detection of Bone Tumors Around the Knee in X-Rays of Children. J. Clin. Med..

[10] Cuocolo R., Caruso M., Perillo T., Ugga L., Petretta M. (2020). Machine Learning in Oncology: A Clinical Appraisal. Cancer Lett..

[11] Martin-Gonzalez P., Crispin-Ortuzar M., Rundo L., Delgado-Ortet M., Reinius M., Brenton J.D., Jimenez-Linan M., Sala E. (2020). Integrative Radiogenomics for Virtual Biopsy and Treatment Monitoring in Ovarian Cancer. Insights Imaging.

[12] Thomford N.E., Bope C.D., Agamah F.E., Dzobo K., Owusu Ateko R., Chimusa E., Mazandu G.K., Ntumba S.B., Dandara C., Wonkam A. (2020). Implementing Artificial Intelligence and Digital Health in Resource-Limited Settings? Top 10 Lessons We Learned in Congenital Heart Defects and Cardiology. OMICS.

[13] Anagnostopoulos A.K., Gaitanis A., Gkiozos I., Athanasiadis E.I., Chatziioannou S.N., Syrigos K.N., Thanos D., Chatziioannou A.N., Papanikolaou N. (2022). Radiomics/Radiogenomics in Lung Cancer: Basic Principles and Initial Clinical Results. Cancers.

[14] Kelly C.J., Karthikesalingam A., Suleyman M., Corrado G., King D. (2019). Key Challenges for Delivering Clinical Impact with Artificial Intelligence. BMC Med..

[15] Tseng H.H., Wei L., Cui S., Luo Y., Ten Haken R.K., El Naqa I. (2020). Machine Learning and Imaging Informatics in Oncology. Oncology.

[16] Saxena S., Jena B., Gupta N., Das S., Sarmah D., Bhattacharya P., Nath T., Paul S., Fouda M.M., Kalra M. (2022). Role of Artificial Intelligence in Radiogenomics for Cancers in the Era of Precision Medicine. Cancers.

[17] Rudie J.D., Rauschecker A.M., Bryan R.N., Davatzikos C., Mohan S. (2019). Emerging Applications of Artificial Intelligence in Neuro-Oncology. Radiology.

[18] Chen B.T., Chen Z., Ye N., Mambetsariev I., Fricke J., Daniel E., Wang G., Wong C.W., Rockne R.C., Colen R.R. (2020). Differentiating Peripherally-Located Small Cell Lung Cancer from Non-Small Cell Lung Cancer Using a CT Radiomic Approach. Front. Oncol..

[19] Mun S.K., Wong K.H., Lo S.B., Li Y., Bayarsaikhan S. (2020). Artificial Intelligence for the Future Radiology Diagnostic Service. Front. Mol. Biosci..

[20] Shiri I., Maleki H., Hajianfar G., Abdollahi H., Ashrafinia S., Hatt M., Zaidi H., Oveisi M., Rahmim A. (2020). Next-Generation Radiogenomics Sequencing for Prediction of EGFR and KRAS Mutation Status in NSCLC Patients Using Multimodal Imaging and Machine Learning Algorithms. Mol. Imaging Biol..

[21] Tu S.J., Wang C.W., Pan K.T., Wu Y.C., Wu C.T. (2018). Localized Thin-Section CT with Radiomics Feature Extraction and Machine Learning to Classify Early-Detected Pulmonary Nodules from Lung Cancer Screening. Phys. Med. Biol..

[22] Ferreira Junior J.R., Koenigkam-Santos M., Cipriano F.E.G., Fabro A.T., Azevedo-Marques P.M. (2018). Radiomics-Based Features for Pattern Recognition of Lung Cancer Histopathology and Metastases. Comput. Methods Programs Biomed..

[23] Bourbonne V., Vallieres M., Lucia F., Doucet L., Visvikis D., Tixier F., Le Prise E., Hatt M., Schick U. (2022). MRI-Derived Radiomics to Guide Post-Operative Management for High-Risk Prostate Cancer Patients. Cancers.

[24] Bourbonne V., Fournier G., Vallières M., Lucia F., Doucet L., Tissot V., Cuvelier C., Hue S., Le Prisé E., Blais E. (2020). External Validation of a Radiomic Signature to Predict p16 Status and Outcome in Oropharyngeal Cancers. Radiother. Oncol..

[25] Grochans S., Cybulska A.M., Simińska D., Korbecki J., Kojder K., Chlubek D., Baranowska-Bosiacka I. (2022). Epidemiology of Glioblastoma Multiforme—Literature Review. Cancers.

[26] Fanizzi A., Catino A., Bove S., Comes M.C., Montrone M., Sicolo A., Signorile R., Perrotti P., Pizzutilo P., Galetta D. (2024). Transfer Learning Approach in Pre-Treatment CT Images to Predict Therapeutic Response in Advanced Malignant Pleural Mesothelioma. Front. Oncol..

[27] Lu I.N., Dobersalske C., Rauschenbach L., Teuber-Hanselmann S., Steinbach A., Ullrich V., Prasad S., Blau T., Kebir S., Siveke J.T. (2021). Tumor-Associated Hematopoietic Stem and Progenitor Cells Positively Linked to Glioblastoma Progression. Nat. Commun..

[28] Kim H.J., Park J.W., Lee J.H. (2021). Genetic Architectures and Cell-of-Origin in Glioblastoma. Front. Oncol..

[29] Melhem J.M., Detsky J., Lim-Fat M.J., Perry J.R. (2022). Updates in IDH-Wildtype Glioblastoma. Neurotherapeutics.

[30] Tykocki T., Eltayeb M. (2018). Ten-Year Survival in Glioblastoma. A Systematic Review. J. Clin. Neurosci..

[31] Jena B., Saxena S., Nayak G.K., Balestrieri A., Gupta N., Khanna N.N., Laird J.R., Kalra M.K., Fouda M.M., Saba L. (2022). Brain Tumor Characterization Using Radiogenomics in Artificial Intelligence Framework. Cancers.

[32] Wikimedia Commons File:Cancer Biomarker Figure.png. Published 30 January 2024. https://commons.wikimedia.org/w/index.php?title=File:Cancer_biomarker_figure.png&oldid=847271038.

[33] Vogrin M., Trojner T., Kelc R. (2020). Artificial Intelligence in Musculoskeletal Oncological Radiology. Radiol. Oncol..

[34] Banerjee I., Crawley A., Bhethanabotla M., Daldrup-Link H.E., Rubin D.L. (2018). Transfer Learning on Fused Multiparametric MR Images for Classifying Histopathological Subtypes of Rhabdomyosarcoma. Comput. Med. Imaging Graph..

[35] Haubold J., Hosch R., Parmar V., Glas M., Guberina N., Catalano O.A., Pierscianek D., Wrede K., Deuschl C., Forsting M. (2023). Fully Automated MR Based Virtual Biopsy of Cerebral Gliomas. Cancers.

[36] Ma F., Shao X., Zhang Y., Li J., Li Q., Sun H., Wang T., Liu H., Zhao F., Chen L. (2024). An Arterial Spin Labeling-Based Radiomics Signature and Machine Learning for the Prediction and Detection of Various Stages of Kidney Damage Due to Diabetes. Front. Endocrinol..

[37] Feng Z., Kong D., Jin W., He K., Zhao J., Liu B., Xu H., Yu X., Feng S. (2023). Rapid Detection of Isocitrate Dehydrogenase 1 Mutation Status in Glioma Based on Crispr-Cas12a. Sci. Rep..

[38] Nagy M., Radakovich N., Nazha A. (2020). Machine Learning in Oncology: What Should Clinicians Know?. JCO Clin. Cancer Inform..

[39] D’Angelo T., Caudo D., Blandino A., Albrecht M.H., Vogl T.J., Gruenewald L.D., Gaeta M., Yel I., Koch V., Martin S.S. (2022). Artificial Intelligence, Machine Learning and Deep Learning in Musculoskeletal Imaging: Current Applications. J. Clin. Ultrasound.

[40] Scapicchio C., Gabelloni M., Barucci A., Cioni D., Saba L., Neri E. (2021). A Deep Look into Radiomics. Radiol. Med..

[41] Pitarch C., Ungan G., Julià-Sapé M., Vellido A. (2024). Advances in the Use of Deep Learning for the Analysis of Magnetic Resonance Image in Neuro-Oncology. Cancers.

[42] Aneja S., Chang E., Omuro A. (2019). Applications of Artificial Intelligence in Neuro-Oncology. Curr. Opin. Neurol..

[43] Shimizu H., Nakayama K.I. (2020). Artificial Intelligence in Oncology. Cancer Sci..

[44] Kawaguchi R.K., Takahashi M., Miyake M., Kinoshita M., Takahashi S., Ichimura K., Hamamoto R., Narita Y., Sese J. (2021). Assessing Versatile Machine Learning Models for Glioma Radiogenomic Studies across Hospitals. Cancers.

[45] Paladugu P.S., Ong J., Nelson N., Kamran S.A., Waisberg E., Zaman N., Kumar R., Dias R.D., Lee A.G., Tavakkoli A. (2023). Generative Adversarial Networks in Medicine: Important Considerations for this Emerging Innovation in Artificial Intelligence. Ann. Biomed. Eng..

[46] Bukowski M., Farkas R., Beyan O., Moll J., Hahn H., Kiessling F., Schmitz-Rode T. (2020). Implementation of eHealth and AI Integrated Diagnostics with Multidisciplinary Digitized Data: Are We Ready from an International Perspective?. Eur. Radiol..

[47] Chang Y., Park H., Yang H.J., Lee S., Lee K.Y., Kim T.S., Shin J., Nam D.H. (2018). Cancer Drug Response Profile Scan (CDRscan): A Deep Learning Model That Predicts Drug Effectiveness from Cancer Genomic Signature. Sci. Rep..

[48] Ciriello G., Miller M.L., Aksoy B.A., Senbabaoglu Y., Schultz N., Sander C. (2013). Emerging Landscape of Oncogenic Signatures across Human Cancers. Nat. Genet..

[49] Cruz J.A., Wishart D.S. (2006). Applications of Machine Learning in Cancer Prediction and Prognosis. Cancer Inform..

[50] “File:Flowchart of the Standard Radiomics Model.png.” Wikimedia Commons. 9 October 2024. https://commons.wikimedia.org/w/index.php?title=File:Flowchart_of_the_standard_radiomics_model.png&oldid=935994688.

[51] Cancer Pharmacogenomics.png. Wikimedia Commons. Published 30 January 2024. https://commons.wikimedia.org/w/index.php?title=File:Cancer_pharmacogenomics.png&oldid=847271761.

[52] The First and Second Phases of the NIH Human Microbiome Project.png. Wikimedia Commons. Published 8 November 2024. https://commons.wikimedia.org/w/index.php?title=File:The_first_and_second_phases_of_the_NIH_Human_Microbiome_Project.png&oldid=954301671.

[53] MultiLayerNeuralNetworkBigger english.png. Wikimedia Commons. Published 15 July 2024. https://commons.wikimedia.org/w/index.php?title=File:MultiLayerNeuralNetworkBigger_english.png&oldid=898886601.

[54] Liu Z., Duan T., Zhang Y., Weng S., Xu H., Ren Y., Zhang Z., Han X. (2023). Radiogenomics: A key component of precision cancer medicine. Br. J. Cancer.

[55] Singh G., Singh A., Bae J., Manjila S., Spektor V., Prasanna P., Lignelli A. (2024). New Frontiers in Domain-Inspired Radiomics and Radiogenomics: Increasing Role of Molecular Diagnostics in CNS Tumor Classification and Grading Following WHO CNS-5 Updates. Cancer Imaging.

[56] Zhang J., Hao L., Qi M., Xu Q., Zhang N., Feng H., Shi G. (2023). Radiomics Nomogram for Preoperative Differentiation of Pulmonary Mucinous Adenocarcinoma from Tuberculoma in Solitary Pulmonary Solid Nodules. BMC Cancer.

[57] Chen Q., Li Y., Cheng Q., Van Valkenburgh J., Sun X., Zheng C., Zhang R., Yuan R. (2022). EGFR Mutation Status and Subtypes Predicted by CT-Based 3D Radiomic Features in Lung Adenocarcinoma. OncoTargets Ther..

[58] Jiang Y., Gao C., Shao Y., Lou X., Hua M., Lin J., Wu L., Gao C. (2024). The Prognostic Value of Radiogenomics Using CT in Patients with Lung Cancer: A Systematic Review. Insights Imaging.

[59] Miles R.C., Lehman C.D., Mercaldo S.F., Tamimi R.M., Hong D., Baker J., McCarthy A.M. (2023). A Radiomics Model for Predicting the Response to Bevacizumab in Brain Necrosis after Nasopharyngeal Carcinoma Radiotherapy. Clin. Transl. Radiat. Oncol..

[60] Spraker M.B., Wootton L.S., Hippe D.S., Ball K.C., Peeken J.C., Macomber M.W., Chapman T.R., Hoff M.N., Kim E.Y., Pollack S.M. (2019). MRI Radiomic Features Are Independently Associated with Overall Survival in Soft Tissue Sarcoma. Adv. Radiat. Oncol..

[61] Wu Y., Xu L., Yang L., Hong J., Zhang Z., Xu Z., Zhou Y., Liu Z., Xu X., Wu D. (2018). Survival Prediction in High-Grade Osteosarcoma Using Radiomics of Diagnostic Computed Tomography. EBioMedicine.

[62] Ngiam K.Y., Khor I.W. (2019). Big Data and Machine Learning Algorithms for Health-Care Delivery. Lancet Oncol..

[63] Lu S.C., Swisher C.L., Chung C., Jaffray D., Sidey-Gibbons C. (2023). On the Importance of Interpretable Machine Learning Predictions to Inform Clinical Decision Making in Oncology. Front. Oncol..

[64] Wang F.A., Li Y., Zeng T. (2024). Deep Learning of Radiology-Genomics Integration for Computational Oncology: A Mini Review. Comput. Struct. Biotechnol. J..

[65] Shams A. (2024). Leveraging State-of-the-Art AI Algorithms in Personalized Oncology: From Transcriptomics to Treatment. Diagnostics.

[66] Kalidindi S. (2024). The Role of Artificial Intelligence in the Diagnosis of Melanoma. Cureus.

[67] Ma J., Song Y., Tian X., Hua Y., Zhang R., Wu J. (2020). Survey on Deep Learning for Pulmonary Medical Imaging. Front. Med..

[68] Paranavithana I.R., Stirling D., Ros M., Field M. (2023). Systematic Review of Tumor Segmentation Strategies for Bone Metastases. Cancers.

[69] Bach Cuadra M., Favre J., Omoumi P. (2020). Quantification in Musculoskeletal Imaging Using Computational Analysis and Machine Learning: Segmentation and Radiomics. Semin. Musculoskelet. Radiol..

[70] Debs P., Fayad L.M. (2023). The Promise and Limitations of Artificial Intelligence in Musculoskeletal Imaging. Front. Radiol..

[71] Montin E., Kijowski R., Youm T., Lattanzi R. (2023). A Radiomics Approach to the Diagnosis of Femoroacetabular Impingement. Front. Radiol..

[72] Gohla G., Kraus M.S., Peyker I., Springer F., Keller G. (2023). Diagnostic Accuracy of 128-Slice Single-Source CT for the Detection of Dislocated Bucket Handle Meniscal Tears in the Setting of an Acute Knee Trauma—Correlation with MRI and Arthroscopy. Diagnostics.

[73] Huber F.A., Guggenberger R. (2022). AI MSK Clinical Applications: Spine Imaging. Skeletal Radiol..

[74] Razavian N., Knoll F., Geras K.J. (2020). Artificial Intelligence in Musculoskeletal Imaging: A Paradigm Shift. Semin. Musculoskelet. Radiol..

[75] Smith S.J., Moorin R., Taylor K., Newton J., Smith S. (2024). Collecting Routine and Timely Cancer Stage at Diagnosis by Implementing a Cancer Staging Tiered Framework: The Western Australian Cancer Registry Experience. BMC Health Serv. Res..

[76] Feuerecker B., Heimer M.M., Geyer T., Fabritius M.P., Gu S., Schachtner B., Beyer L., Ricke J., Gatidis S., Ingrisch M. (2023). Artificial Intelligence in Oncological Hybrid Imaging. Rofo.

[77] D’Amore B., Smolinski-Zhao S., Daye D., Uppot R.N. (2021). Role of Machine Learning and Artificial Intelligence in Interventional Oncology. Curr. Oncol. Rep..

[78] Di J., Hickey C., Bumgardner C., Yousif M., Zapata M., Bocklage T., Balzer B., Bui M.M., Gardner J.M., Pantanowitz L. (2024). Utility of artificial intelligence in a binary classification of soft tissue tumors. J. Pathol Inform..

[79] Salehi M.A., Mohammadi S., Harandi H., Zakavi S.S., Jahanshahi A., Shahrabi Farahani M., Wu J.S. (2024). Diagnostic Performance of Artificial Intelligence in Detection of Primary Malignant Bone Tumors: A Meta-Analysis. J. Imaging Inform. Med..

[80] Ong W., Zhu L., Tan Y.L., Teo E.C., Tan J.H., Kumar N., Vellayappan B.A., Ooi B.C., Quek S.T., Makmur A. (2023). Application of Machine Learning for Differentiating Bone Malignancy on Imaging: A Systematic Review. Cancers.

[81] Choi J.H., Ro J.Y. (2023). The Recent Advances in Molecular Diagnosis of Soft Tissue Tumors. Int. J. Mol. Sci..

[82] Bi W.L., Hosny A., Schabath M.B., Giger M.L., Birkbak N.J., Mehrtash A., Allison T., Arnaout O., Abbosh C., Dunn I.F. (2019). Artificial intelligence in cancer imaging: Clinical challenges and applications. CA Cancer J. Clin..

[83] Schajowicz F., McGuire M.H. (1989). Diagnostic difficulties in skeletal pathology. Clin. Orthop. Relat. Res..

[84] van Griethuysen J.J.M., Fedorov A., Parmar C., Hosny A., Aucoin N., Narayan V., Beets-Tan R.G.H., Fillion-Robin J.C., Pieper S., Aerts H.J.W.L. (2017). Computational Radiomics System to Decode the Radiographic Phenotype. Cancer Res..

[85] Lambin P., Rios-Velazquez E., Leijenaar R., Carvalho S., van Stiphout R.G.P.M., Granton P., Zegers C.M.L., Gillies R., Boellard R., Dekker A. (2012). Radiomics: Extracting More Information from Medical Images Using Advanced Feature Analysis. Eur. J. Cancer.

[86] Mayerhoefer M.E., Materka A., Langs G., Häggström I., Szczypiński P., Gibbs P., Cook G. (2020). Introduction to Radiomics. J. Nucl. Med..

[87] Gillies R.J., Kinahan P.E., Hricak H. (2016). Radiomics: Images Are More than Pictures, They Are Data. Radiology.

[88] Shur J.D., Doran S.J., Kumar S., Ap Dafydd D., Downey K., O’Connor J.P.B., Poptani H., Koh D.M., Orton M.R., Messiou C. (2021). Radiomics in Oncology: A Practical Guide. Radiographics.

[89] Kocak B., Durmaz E.S., Ates E., Kilickesmez O. (2021). Radiomics with Artificial Intelligence: A Review for the Nonradiologist. Diagn. Interv. Radiol..

[90] Cè M., Caloro E., Pellegrino M.E., Basile M., Sorce A., Fazzini D., Oliva G., Cellina M. (2022). Artificial Intelligence in Breast Cancer Imaging: Risk Stratification, Lesion Detection and Classification, Treatment Planning and Prognosis—A Narrative Review. Explor. Target. Antitumor Ther..

[91] Liu Z., Wang S., Dong D., Wei J., Fang C., Zhou X., Sun K., Li L., Li B., Wang M. (2019). The Applications of Radiomics in Precision Diagnosis and Treatment of Oncology: Opportunities and Challenges. Theranostics.

[92] Park J.E., Kim D., Kim H.S., Park S.Y., Kim J.Y., Cho S.J., Shin J.H., Kim J.H. (2020). Quality of Science and Reporting of Radiomics in Oncologic Studies: Room for Improvement According to Radiomics Quality Score and TRIPOD Statement. Eur. Radiol..

[93] Jiang R., Jiang J., Zhao L., Zhang J., Zhang S., Yao Y., Yang S., Shi J., Shen N., Su C. (2015). Diffusion kurtosis imaging can efficiently assess the glioma grade and cellular proliferation. Oncotarget.

[94] Radiomic Features—Pyradiomics 2.2.0.post35+g8da1db7 Documentation Readthedocs.io. Published 2016..

[95] Rios Velazquez E., Parmar C., Liu Y., Coroller T.P., Cruz G., Stringfield O., Ye Z., Makrigiorgos M., Fennessy F., Mak R.H. (2017). Somatic Mutations Drive Distinct Imaging Phenotypes in Lung Cancer. Cancer Res..

[96] Fusco R., Sansone M., Granata V., Di Bonito M., Avino F., Catalano O., Botti G., Petrillo A. (2018). Use of Quantitative Morphological and Functional Features for Assessment of Axillary Lymph Node in Breast Dynamic Contrast-Enhanced Magnetic Resonance Imaging. Biomed. Res. Int..

[97] Zubair A.R., Alo O.A. (2024). Grey Level Co-occurrence Matrix (GLCM) Based Second Order Statistics for Image Texture Analysis. arXiv.

[98] Aerts H.J.W.L., Velazquez E.R., Leijenaar R.T.H., Parmar C., Grossmann P., Carvalho S., Bussink J., Monshouwer R., Haibe-Kains B., Rietveld D. (2014). Decoding Tumour Phenotype by Noninvasive Imaging Using a Quantitative Radiomics Approach. Nat. Commun..

[99] Yip S.S.F., Aerts H.J.W.L. (2016). Applications and Limitations of Radiomics. Phys. Med. Biol..

[100] Limkin C.J., Sun R., Dercle L., Zacharaki E.I., Robert C., Reuzé S., Schernberg A., Paragios N., Deutsch E., Ferté C. (2017). Promises and Challenges for the Implementation of Computational Medical Imaging (Radiomics) in Oncology. Ann. Oncol..

[101] Rizzo S., Botta F., Raimondi S., Origgi D., Fanciulli M., Morganti A.G., Bellomi M. (2018). Radiomics: The Facts and the Challenges of Image Analysis. Eur. Radiol. Exp..

[102] Leithner D., Horvat J.V., Marino M.A., Bernard-Davila B., Jochelson M.S., Ochoa-Albiztegui R.E., Martinez D.F., Morris E.A., Thakur S., Pinker K. (2019). Radiomic Signatures with Contrast-Enhanced Magnetic Resonance Imaging for the Assessment of Breast Cancer Receptor Status and Molecular Subtypes: Initial Results. Breast Cancer Res..

[103] WBodalal Z., Trebeschi S., Nguyen-Kim T.D.L., Schats W., Beets-Tan R. (2019). Radiogenomics: Bridging Imaging and Genomics. Abdom. Radiol..

[104] Winfield J.M., Miah A.B., Strauss D., Thway K., Collins D.J., deSouza N.M., Leach M.O., Morgan V.A., Giles S.L., Moskovic E. (2019). Utility of Quantitative Magnetic Resonance Imaging Parameters in the Evaluation of Soft Tissue Sarcomas. J. Magn. Reson. Imaging.

[105] Crombé A., Périer C., Kind M., De Senneville B.D., Le Loarer F., Italiano A., Buy X., Saut O. (2019). T2-Based MRI Delta-Radiomics Improve Response Prediction in Soft-Tissue Sarcomas Treated by Neoadjuvant Chemotherapy. J. Magn. Reson. Imaging.

[106] Vallières M., Freeman C.R., Skamene S.R., El Naqa I. (2015). A Radiomics Model from Joint FDG-PET and MRI Texture Features for the Prediction of Lung Metastases in Soft-Tissue Sarcomas of the Extremities. Phys. Med. Biol..

[107] Nakajo M., Kobayashi H., Natsume T., Shinohara T., Fukukura Y., Fujisaki Y., Hirahara Y., Nakajo M., Yoshiura T. (2018). Dual-Energy CT-Derived Iodine Content and Spectral Attenuation Analysis of Metastatic Versus Nonmetastatic Lymph Nodes in Squamous Cell Carcinoma of the Oropharynx. Tomography..

[108] Gaillard F. Hounsfield Unit Formula. Case Study, Radiopaedia.org.

[109] Peeken J.C., Bernhofer M., Wiestler B., Goldberg T., Cremers D., Rost B., Weber M.A., Diehl C.D., Zimmer C., Combs S.E. (2018). Radiomics in Radiooncology—Challenging the Medical Physicist. Phys. Med..

[110] Long H., Zhang P., Bi Y., Yang C., Wu M., He D., Huang S., Yang K., Qi S., Wang J. (2023). MRI Radiomic Features of Peritumoral Edema May Predict the Recurrence Sites of Glioblastoma Multiforme. Front. Oncol..

[111] Gao M., Huang S., Pan X., Liao X., Yang R., Zhou G., Liu Y., Yang Z., Liu J., Qin G. (2023). Machine Learning-Based Radiomics for Prediction of Epidermal Growth Factor Receptor Mutations in Lung Adenocarcinoma. J. Comput. Assist. Tomogr..

[112] Kumar R., Sporn K., Ong J., Waisberg E., Paladugu P., Vaja S., Hage T., Sekhar T.C., Vadhera A.S., Ngo A. (2025). Integrating Artificial Intelligence in Orthopedic Care: Advancements in Bone Care and Future Directions. Bioengineering.

[113] De Angelis R., Casale R., Coquelet N., Ikhlef S., Mokhtari A., Simoni P., Bali M.A. (2024). The Impact of Radiomics in the Management of Soft Tissue Sarcoma. Discov. Oncol..

[114] Bailly C., Bodet-Milin C., Couespel S., Necib H., Kraeber-Bodéré F., Ansquer C., Carlier T. (2016). Revisiting the Robustness of PET-Based Textural Features in the Context of Multi-Centric Trials. PLoS ONE.

[115] Lovinfosse P., Polus M., Van Daele D., Martinive P., Daenen F., Hatt M., Visvikis D., Hustinx R., Withofs N. (2018). FDG PET/CT Radiomics for Predicting the Outcome of Locally Advanced Rectal Cancer. Eur. J. Nucl. Med. Mol. Imaging.

[116] Larson S.M., Erdi Y., Akhurst T., Mazumdar M., Macapinlac H.A., Finn R.D., Casilla C., Fazzari M., Srivastava N., Yeung H.W.D. (1999). Tumor treatment response based on visual and quantitative changes in global tumor glycolysis using PET-FDG imaging. The visual response score and the change in total lesion glycolysis. Clin. Positron Imaging.

[117] Im H.J., Bradshaw T., Solaiyappan M., Cho S.Y. (2018). Current Methods to Define Metabolic Tumor Volume in Positron Emission Tomography: Which One is Better?. Nucl. Med. Mol. Imaging.

[118] Ha S., Choi H., Paeng J.C., Cheon G.J. (2019). Radiomics in Oncological PET/CT: A Methodological Overview. Nucl. Med. Mol. Imaging.

[119] Suzuki H., Nishio M., Nakanishi H., Hanai N., Hirakawa H., Kodaira T., Tamaki T., Hasegawa Y. (2016). Impact of total lesion glycolysis measured by 18F-FDG-PET/CT on overall survival and distant metastasis in hypopharyngeal cancer. Oncol Lett..

[120] Orlhac F., Boughdad S., Philippe C., Stalla-Bourdillon H., Nioche C., Champion L., Soussan M., Frouin F., Frouin V., Buvat I. (2018). A Postreconstruction Harmonization Method for Multicenter Radiomic Studies in PET. J. Nucl. Med..

[121] Sun R., Orlhac F., Robert C., Reuzé S., Schernberg A., Buvat I., Deutsch E., Ferté C. (2016). In Regard to Mattonen et al. Int. J. Radiat. Oncol. Biol. Phys..

[122] Traverso A., Wee L., Dekker A., Gillies R. (2018). Repeatability and Reproducibility of Radiomic Features: A Systematic Review. Int. J. Radiat. Oncol. Biol. Phys..

[123] Kumar V., Gu Y., Basu S., Berglund A., Eschrich S.A., Schabath M.B., Forster K., Aerts H.J.W.L., Dekker A., Fenstermacher D. (2012). Radiomics: The Process and the Challenges. Magn. Reson. Imaging.

[124] File:TORNAI-SpectrumOfMedicalImaging.jpg Wikimedia Commons. Published 5 November 2024. https://commons.wikimedia.org/w/index.php?title=File:TORNAI-SpectrumOfMedicalImaging.jpg&oldid=953058884.

[125] Parmar C., Grossmann P., Bussink J., Lambin P., Aerts H.J.W.L. (2015). Machine Learning Methods for Quantitative Radiomic Biomarkers. Sci. Rep..

[126] Leger S., Zwanenburg A., Pilz K., Lohaus F., Linge A., Zöphel K., Kotzerke J., Schreiber A., Tissot V., Dreveau C. (2017). A Comparative Study of Machine Learning Methods for Time-to-Event Survival Data for Radiomics Risk Modelling. Sci. Rep..

[127] Lee J.Y., Lee K.S., Seo B.K., Cho K.R., Woo O.H., Song S.E., Kim E.K., Lee H.Y., Kim J.S., Cha J. (2022). Radiomic Machine Learning for Predicting Prognostic Biomarkers and Molecular Subtypes of Breast Cancer Using Tumor Heterogeneity and Angiogenesis Properties on MRI. Eur. Radiol..

[128] Chen R.J., Lu M.Y., Chen T.Y., Williamson D.F.K., Mahmood F. (2021). Synthetic Data in Machine Learning for Medicine and Healthcare. Nat. Biomed. Eng..

[129] HajiEsmailPoor Z., Tabnak P., Baradaran B., Pashazadeh F., Aghebati-Maleki L. (2023). Diagnostic Performance of CT Scan-Based Radiomics for Prediction of Lymph Node Metastasis in Gastric Cancer: A Systematic Review and Meta-Analysis. Front. Oncol..

[130] Stanzione A., Cuocolo R., Ugga L., Verde F., Romeo V., Mainenti P.P., Brunetti A., Maurea S. (2022). Radiomics in Cross-Sectional Adrenal Imaging: A Systematic Review and Quality Assessment Study. Diagnostics.

[131] Zwanenburg A., Vallières M., Abdalah M.A., Aerts H.J.W.L., Andrearczyk V., Apte A., Ashrafinia S., Bakas S., Beukinga R.J., Boellaard R. (2020). The Image Biomarker Standardization Initiative: Standardized Quantitative Radiomics for High-Throughput Image-Based Phenotyping. Radiology.

[132] Orlhac F., Frouin F., Nioche C., Ayache N., Buvat I. (2019). Validation of A Method to Compensate Multicenter Effects Affecting CT Radiomics. Radiology.

[133] Da-Ano R., Visvikis D., Hatt M. (2020). Harmonization Strategies for Multicenter Radiomics Investigations. Phys. Med. Biol..

[134] Scalco E., Rizzo G. (2020). Texture Analysis of Medical Images in Radiomics: Review. Phys. Med..

[135] Berenguer R., Pastor-Juan M.D.R., Canales-Vázquez J., Castro-García M., Villas M.V., Mansilla Legorburo F., Sabaté-Llobera A. (2018). Radiomics of CT Features May Be Nonreproducible and Redundant: Influence of CT Acquisition Parameters. Radiology.

[136] Schwier M., van Griethuysen J., Vangel M.G., Pieper S., Peled S., Tempany C., Aerts H.J.W.L., Kikinis R., Fennessy F.M., Fedorov A. (2019). Repeatability of Multiparametric Prostate MRI Radiomics Features. Sci. Rep..

[137] Park B.W., Kim J.K., Lim H.J., Park J.J., Lee J., Byeon J.S., Park H., Kim Y., Lee E.J., Oh C. (2021). Temporal Changes of Quantitative CT Findings from 102 Patients with COVID-19 in Wuhan, China: A Longitudinal Study. J. Korean Med. Sci..

[138] “File:K-fold Cross Validation EN.svg.” Wikimedia Commons. 3 October 2024. https://commons.wikimedia.org/w/index.php?title=File:K-fold_cross_validation_EN.svg&oldid=932198002.

[139] van Timmeren J.E., Cester D., Tanadini-Lang S., Alkadhi H., Baessler B. (2020). Radiomics in Medical Imaging—“How-To” Guide and Critical Reflection. Insights Imaging.

[140] Jha A.K., Mithun S., Sherkhane U.B., Dwivedi P., Puts S., Osong B., Traverso A., Purandare N., Wee L., Rangarajan V. (2023). Emerging Role of Quantitative Imaging (Radiomics) and Artificial Intelligence in Precision Oncology. Explor. Target. Antitumor Ther..

[141] Ibrahim A., Primakov S., Beuque M., Woodruff H.C., Halilaj I., Wu G., Refaee T., Granzier R., Widaatalla Y., Hustinx R. (2021). Radiomics for Precision Medicine: Current Challenges, Future Prospects, and the Proposed 6Rs Framework. Methods.

[142] Zhao B., Tan Y., Tsai W.Y., Qi J., Xie C., Lu L., Schwartz L.H. (2016). Reproducibility of Radiomics for Deciphering Tumor Phenotype with Imaging. Sci. Rep..

[143] Altazi B.A., Zhang G.G., Fernandez D.C., Montejo M.E., Hunt D., Werner J., Biagioli M.C., Moros E.G. (2019). Reproducibility and Generalizability in Radiomics Modeling: Possible Strategies in Radiologic and Statistical Modeling. BJR Open.

[144] Tixier F., Um H., Bermudez D., Iyer A., Apte A., Graham M.S., Neff C.M., Sutton E.J., Deasy J.O., Hatzoglou V. (2019). Preoperative MRI-Radiomics Features Improve Prediction of Survival in Glioblastoma Patients over MGMT Methylation Status Alone. Oncotarget.

[145] Fornacon-Wood I., Faivre-Finn C., O’Connor J.P.B., Price G.J. (2022). Radiomics as a Personalized Medicine Tool in Lung Cancer: Predicting Treatment Response from Baseline Imaging. Clin. Lung Cancer.

[146] “File:Real-to-Voxel-Intensity-Range.png.” Wikimedia Commons. 12 May 2024. https://commons.wikimedia.org/w/index.php?title=File:Real-to-Voxel-intensity-range.png&oldid=876059423.

[147] Zwanenburg A., Leger S., Vallières M., Löck S. (2016). For the Image Biomarker Standardization Initiative. Image Biomarker Standardisation Initiative. arXiv.

[148] Pati S., Singh A., Rathore S., Gastounioti A., Bergman M., Ngo P., Ha S.M., Bounias D., Minock J., Murphy G. (2020). The Cancer Imaging Phenomics Toolkit (CaPTk): Technical Overview. Brainlesion.

[149] Chalkidou A., O’Doherty M.J., Marsden P.K. (2015). False Discovery Rates in PET and CT Studies with Texture Features: A Systematic Review. PLoS ONE.

[150] Sauerbrei W., Perperoglou A., Schmid M., Abrahamowicz M., Becher H., Binder H., Dunkler D., Harrell F.E., Royston P., Heinze G. (2020). State of the Art in Selection of Variables and Functional Forms in Multivariable Analysis—Outstanding Issues. Diagn. Progn. Res..

[151] Welch M.L., McIntosh C., Haibe-Kains B., Milosevic M.F., Wee L., Dekker A., Huang S.H., Purdie T.G., O’Sullivan B., Aerts H.J.W.L. (2019). Vulnerabilities of Radiomic Signature Development: The need for safeguards. Radiother Oncol..

[152] Abeshouse A., Adebamowo C., Adebamowo S.N., Akbani R., Akeredolu T., Ally A., Anderson M.L., Anur P., Appelbaum E.L., Armenia J. (2017). Comprehensive and Integrated Genomic Characterization of Adult Soft Tissue Sarcomas. Cell.

[153] D’Angelo S.P., Antonescu C.R. (2021). Targeted Therapies for Sarcomas: New Frontiers in Precision Medicine. J. Mol. Diagn..

[154] Sayles L.C., Breese M.R., Koehne A.L., Leung S.G., Lee A.G., Liu H.Y., Spillinger A., Shah A.T., Tanasa B., Straessler K.M. (2019). Genome-Informed Targeted Therapy for Osteosarcoma. Cancer Discov..

[155] Sheffield N.C., Pierron G., Klughammer J., Datlinger P., Schönegger A., Schuster M., Hadler J., Surdez D., Guillemot D., Lapouble E. (2017). DNA Methylation Heterogeneity Defines a Disease Spectrum in Ewing Sarcoma. Nat. Med..

[156] Chen X., Bahrami A., Pappo A., Easton J., Dalton J., Hedlund E., Ellison D., Shurtleff S., Wu G., Wei L. (2014). Recurrent Somatic Structural Variations Contribute to Tumorigenesis in Pediatric Osteosarcoma. Cell Rep..

[157] Sadikovic B., Yoshimoto M., Chilton-MacNeill S., Thorner P., Squire J.A., Zielenska M. (2009). Identification of Interactive Networks of Gene Expression Associated with Osteosarcoma Oncogenesis by Integrated Molecular Profiling. Hum. Mol. Genet..

[158] Wu C.C., Beird H.C., Andrew Livingston J., Advani S., Akdemir K.C., Mitra A., Kim S., Ingram D.R., Wang W.L., Lazar A.J. (2020). Immuno-Genomic Landscape of Osteosarcoma. Front. Immunol..

[159] Riggi N., Knoechel B., Gillespie S.M., Rheinbay E., Boulay G., Suvà M.L., Rossetti N.E., Boonseng W.E., Oksuz O., Cook E.B. (2014). EWS-FLI1 Utilizes Divergent Chromatin Remodeling Mechanisms to Directly Activate or Repress Enhancer Elements in Ewing Sarcoma. Cancer Cell.

[160] Gorkin D.U., Barozzi I., Zhao Y., Zhang Y., Huang H., Lee A.Y., Li B., Chiou J., Wildberg A., Ding B. (2020). An Atlas of Dynamic Chromatin Landscapes in Mouse Fetal Development. Nature.

[161] Orrapin S., Moonmuang S., Udomruk S., Yongpitakwattana P., Pruksakorn D., Chaiyawat P. (2024). Unlocking the Tumor-Immune Microenvironment in Osteosarcoma: Insights into the Immune Landscape and Mechanisms. Front. Immunol..

[162] Tatsuno R., Komohara Y., Pan C., Kawasaki T., Enomoto A., Jubashi T., Kono H., Wako M., Ashizawa T., Haro H. (2024). Surface Markers and Chemokines/Cytokines of Tumor-Associated Macrophages in Osteosarcoma and Other Carcinoma Microenvironments—Contradictions and Comparisons. Cancers.

[163] Gong L., Sun X., Jia M. (2023). New Gene Signature from the Dominant Infiltration Immune Cell Type in Osteosarcoma Predicts Overall Survival. Sci. Rep..

[164] Zhou J., Zhao L., Xiao Y., Xie S., Long Y., Wei Y., Meng Q., Li X., Luo H., Zhu H. (2022). The Expression of Cytokine Profiles and Related Receptors in Idiopathic Inflammatory Myopathies. Front. Pharmacol..

[165] Kany S., Vollrath J.T., Relja B. (2019). Cytokines in Inflammatory Disease. Int. J. Mol. Sci..

[166] Kartikasari A.E.R., Huertas C.S., Mitchell A., Plebanski M. (2021). Tumor-Induced Inflammatory Cytokines and the Emerging Diagnostic Devices for Cancer Detection and Prognosis. Front. Oncol..

[167] Italiano A., Mathoulin-Pelissier S., Le Cesne A., Terrier P., Bonvalot S., Collin F., Michels J.J., Blay J.Y., Coindre J.M., Bui B.N. (2011). Trends in Survival for Patients with Metastatic Soft-Tissue Sarcoma. Cancer.

[168] Davis L.E., Jeng S., Svalina M.N., Huang E., Pittsenbarger J., Cantor E.L., Berlow N., Seguin B., Mansoor A., McWeeney S.K. (2017). Integration of genomic, transcriptomic and functional profiles of aggressive osteosarcomas across multiple species. Oncotarget.

[169] Frazzette N., Jour G. (2025). Novel Molecular Methods in Soft Tissue Sarcomas: From Diagnostics to Theragnostics. Cancers.

[170] Zou Z., Sun W., Xu Y., Liu W., Zhong J., Lin X., Chen Y. (2022). Application of Multi-Omics Approach in Sarcomas: A Tool for Studying Mechanism, Biomarkers, and Therapeutic Targets. Front. Oncol..

[171] Gooding S., Wang C.-Y., Baker W., Watson E., Davis S., Fischer R., McCallion O., Hester J., Issa F., Rao S. (2024). Development of a Spatial Multiomics Platform to Integrate Genomic, Transcriptomic and Proteomic Features for Translational Research in Multiple Myeloma. Blood.

[172] He W., Huang W., Zhang L., Wu X., Zhang S., Zhang B. (2024). Radiogenomics: Bridging the gap between imaging and genomics for precision oncology. MedComm. (2020).

[173] Yoo C., Jeong H., Jeong J.H., Kim K.P., Lee S., Ryoo B.Y., Hwang D.W., Lee J.H., Moon D.B., Kim K.H. (2025). Circulating tumor DNA status and dynamics predict recurrence in patients with resected extrahepatic cholangiocarcinoma. J. Hepatol..

[174] Sharpe M., Mount N. (2015). Genetically Modified T Cells in Cancer Therapy: Opportunities and Challenges. Dis. Model. Mech..

[175] Su G.H., Xiao Y., You C., Zheng R.C., Zhao S., Sun S.Y., Zhou J.Y., Lin L.Y., Wang H., Shao Z.M. (2023). Radiogenomic-Based Multiomic Analysis Reveals Imaging Intratumor Heterogeneity Phenotypes and Therapeutic Targets. Sci. Adv..

[176] Zhou M., Leung A., Echegaray S., Gentles A., Shrager J.B., Jensen K.C., Berry G.J., Plevritis S.K., Rubin D.L., Napel S. (2018). Non-Small Cell Lung Cancer Radiogenomics Map Identifies Relationships between Molecular and Imaging Phenotypes with Prognostic Implications. Radiology.

[177] O’Sullivan N.J., Temperley H.C., Horan M.T., Curtain B.M.M., O’Neill M., Donohoe C., Ravi N., Corr A., Meaney J.F.M., Reynolds J.V. (2024). Computed Tomography (CT) Derived Radiomics to Predict Post-Operative Disease Recurrence in Gastric Cancer: A Systematic Review and Meta-Analysis. Curr. Probl. Diagn. Radiol..

[178] Choi W., Oh J.H., Riyahi S., Liu C.J., Feng F., Chen W., Huang C., Lu W., Saha P.K., Torigian D.A. (2018). Radiologic and Genomic Evolution of Individual Metastases during HER2 Blockade in Colorectal Cancer. Cancer Cell.

[179] Guo X., Deng Y., Jiang W., Li H., Luo Y., Zhang H., Wu H. (2025). Single Cell Transcriptomic Analysis Reveals Tumor Immune Infiltration by Macrophage Cells Gene Signature in Lung Adenocarcinoma. Discov. Oncol..

[180] Spratt D.E., Alshalalfa M., Fishbane N., Weiner A.B., Mehra R., Mahal B.A., Lehrer J., Liu Y., Zhao S.G., Speers C. (2020). Transcriptomic Heterogeneity of Gleason Grade Group 5 Prostate Cancer. Eur. Urol..

[181] Gibault L., Pérot G., Chibon F., Filhine-Trésarrieu P., Laplanche A., Michon J., Coindre J.M., Lagarde P., Terrier P., Mauduit O. (2024). New Insights into the Prognostic Value of Genomic Classification in Synovial Sarcoma. Eur. J. Cancer.

[182] Weigelt B., Vargas H.A., Selenica P., Geyer F.C., Mazaheri Y., Blecua P., Conlon N., Hoang L.N., Jungbluth A.A., Snyder A. (2019). Radiogenomics Analysis of Intratumor Heterogeneity in a Patient with High-Grade Serous Ovarian Cancer. JCO Precis. Oncol..

[183] Gitto S., Cuocolo R., Huisman M., Messina C., Albano D., Omoumi P., Kotter E., Maas M., Van Ooijen P., Sconfienza L.M. (2024). CT and MRI Radiomics of Bone and Soft-Tissue Sarcomas: An Updated Systematic Review of Reproducibility and Validation Strategies. Insights Imaging.

[184] Peeken J.C., Spraker M.B., Knebel C., Dapper H., Pfeiffer D., Devecka M., Thamer A., Shouman M.A., Ott A., von Eisenhart-Rothe R. (2019). Tumor Grading of Soft Tissue Sarcomas Using MRI-Based Radiomics. EBioMedicine.

[185] Xie W., Reder N.P., Koyuncu C., Leo P., Hawley S., Huang H., Mao C., Postupna N., Kang S., Serafin R. (2022). Prostate Cancer Risk Stratification via Nondestructive 3D Pathology with Deep Learning-Assisted Gland Analysis. Cancer Res..

[186] Lafata K.J., Corradetti M.N., Gao J., Jacobs C.D., Weng J., Chang Y., Wang C., Hatch A., Xanthopoulos E., Jones G. (2021). Radiogenomic Analysis of Locally Advanced Lung Cancer Based on CT Imaging and Intratreatment Changes in Cell-Free DNA. Radiol. Imaging Cancer.

[187] Mendes Serrão E., Klug M., Moloney B.M., Jhaveri A., Lo Gullo R., Pinker K., Luker G., Haider M.A., Shinagare A.B., Liu X. (2023). Current Status of Cancer Genomics and Imaging Phenotypes: What Radiologists Need to Know. Radiol. Imaging Cancer.

[188] Yamamoto S., Maki D.D., Korn R.L., Kuo M. (2012). Radiogenomic Analysis of Breast Cancer Using MRI: A Preliminary Study to Define the Landscape. Am. J. Roentgenol..

[189] Fan M., Xia P., Clarke R., Wang Y., Li L. (2020). Radiogenomic signatures reveal multiscale intratumour heterogeneity associated with biological functions and survival in breast cancer. Nat. Commun..

[190] Tian H., Cao J., Li B., Nice E.C., Mao H., Zhang Y., Huang C. (2023). Managing the Immune Microenvironment of Osteosarcoma: The Outlook for Osteosarcoma Treatment. Bone Res..

[191] Zeng J., Wang D., Tong Z., Li Z., Wang G., Du Y., Li J., Miao J., Chen S. (2025). Development of a Prognostic Model for Osteosarcoma Based on Macrophage Polarization-Related Genes Using Machine Learning: Implications for Personalized Therapy. Clin. Exp. Med..

[192] Jeys L.M., Thorne C.J., Parry M., Gaston C.L., Sumathi V.P., Grimer J.R. (2017). A Novel System for the Surgical Staging of Primary High-Grade Osteosarcoma: The Birmingham Classification. Clin. Orthop. Relat. Res..

[193] Bacci G., Longhi A., Versari M., Mercuri M., Briccoli A., Picci P. (2006). Prognostic Factors for Osteosarcoma of the Extremity Treated with Neoadjuvant Chemotherapy: 15-Year Experience in 789 Patients Treated at a Single Institution. Cancer.

[194] Bielack S.S., Kempf-Bielack B., Delling G., Exner G.U., Flege S., Helmke K., Kotz R., Salzer-Kuntschik M., Werner M., Winkelmann W. (2002). Prognostic Factors in High-Grade Osteosarcoma of the Extremities or Trunk: An Analysis of 1,702 Patients Treated on Neoadjuvant Cooperative Osteosarcoma Study Group Protocols. J. Clin. Oncol..

[195] Ferrari S., Bertoni F., Mercuri M., Picci P., Giacomini S., Longhi A., Bacci G. (2001). Predictive Factors of Disease-Free Survival for Non-Metastatic Osteosarcoma of the Extremity: An. Analysis of 300 Patients Treated at the Rizzoli Institute. Ann. Oncol..

[196] Palmerini E., Torricelli E., Cascinu S., Pierini M., De Paolis M., Donati D., Cesari M., Longhi A., Abate M., Paioli A. (2019). Is There a Role for Chemotherapy after Local Relapse in High-Grade Osteosarcoma?. Pediatr. Blood Cancer.

[197] Smeland S., Bielack S.S., Whelan J., Bernstein M., Hogendoorn P., Krailo M.D., Gorlick R., Janeway K.A., Ingleby F.C., Anninga J. (2019). Survival and Prognosis with Osteosarcoma: Outcomes in More than 2000 Patients in the EURAMOS-1 (European and American Osteosarcoma Study) Cohort. Eur. J. Cancer.

[198] Whelan J.S., Jinks R.C., McTiernan A., Sydes M.R., Hook J.M., Trani L., Uscinska B., Bramwell V., Lewis I.J., Nooij M.A. (2012). Survival from High-Grade Localised Extremity Osteosarcoma: Combined Results and Prognostic Factors from Three European Osteosarcoma Intergroup Randomised Controlled Trials. Ann. Oncol..

[199] Strauss S.J., Frezza A.M., Abecassis N., Bajpai J., Bauer S., Biagini R., Bielack S., Blay J.Y., Bolle S., Bonvalot S. (2021). ESMO Guidelines Committee, EURACAN, GENTURIS, and ERN PaedCan. Bone Sarcomas: ESMO–EURACAN–GENTURIS–ERN PaedCan Clinical Practice Guideline for Diagnosis, Treatment and Follow-Up. Ann. Oncol..

[200] Isakoff M.S., Bielack S.S., Meltzer P., Gorlick R. (2015). Osteosarcoma: Current Treatment and a Collaborative Pathway to Success. J. Clin. Oncol..

[201] Chen Y., Gokavarapu S., Shen L., Liu F., Cao W., Ling Y., Ji T. (2017). Chemotherapy in Head and Neck Osteosarcoma: A Retrospective Analysis of 27 Patients from a Single Institution. Oral Oncol..

[202] Fu Y., Xu Y., Liu W., Zhang J., Wang F., Jian Q., Huang G., Zou C., Xie X., Kim A.H. (2024). Tumor-Informed Deep Sequencing of ctDNA Detects Minimal Residual Disease and Predicts Relapse in Osteosarcoma. EClinicalMedicine.

[203] Meltzer P.S., Helman L.J. (2021). New Horizons in the Treatment of Osteosarcoma. N. Engl. J. Med..

[204] Lilienthal I., Herold N. (2020). Targeting Molecular Mechanisms Underlying Treatment Efficacy and Resistance in Osteosarcoma: A Review of Current and Future Strategies. Int. J. Mol. Sci..

[205] Eaton B.R., Schwarz R., Vatner R., Yeh B., Claude L., Indelicato D.J., Laack N. (2021). Osteosarcoma. Pediatr. Blood Cancer.

[206] Ritter J., Bielack S.S. (2010). Osteosarcoma. Ann. Oncol..

[207] Kansara M., Teng M.W., Smyth M.J., Thomas D.M. (2014). Translational Biology of Osteosarcoma. Nat. Rev. Cancer.

[208] File:SVM explain.png Wikimedia Commons. Published 22 February 2022. https://commons.wikimedia.org/w/index.php?title=File:SVM_explain.png&oldid=631507021.

[209] File:Random forest explain.png Wikimedia Commons. Published 20 November 2021. https://commons.wikimedia.org/w/index.php?title=File:Random_forest_explain.png&oldid=608403035.

[210] Anderson M.E. (2016). Update on Survival in Osteosarcoma. Orthop. Clin. N. Am..

[211] Meazza C., Scanagatta P. (2016). Metastatic Osteosarcoma: A Challenging Multidisciplinary Treatment. Expert. Rev. Anticancer Ther..

[212] Harrison D.J., Geller D.S., Gill J.D., Lewis V.O., Gorlick R. (2018). Current and Future Therapeutic Approaches for Osteosarcoma. Expert. Rev. Anticancer Ther..

[213] Kager L., Tamamyan G., Bielack S. (2017). Novel Insights and Therapeutic Interventions for Pediatric Osteosarcoma. Future Oncol..

[214] van Maldegem A.M., Bhosale A., Gelderblom H.J., Hogendoorn P.C., Hassan A.B. (2016). Comprehensive Analysis of Published Phase I/II Clinical Trials for Osteosarcoma and Ewing Sarcoma. Eur. J. Cancer.

[215] Lagmay J.P., Krailo M.D., Dang H., Kim A., Hawkins D.S., Beaty O., Widemann B.C., Zwerdling T., Bomgaars L., Langevin A.M. (2016). Outcome of Patients with Recurrent Osteosarcoma Enrolled in Seven Phase II Trials through Children’s Cancer Group, Pediatric Oncology Group, and Children’s Oncology Group. Pediatr. Blood Cancer.

[216] “File:Feature Learning Diagram.png.” Wikimedia Commons. 9 October 2022. https://commons.wikimedia.org/w/index.php?title=File:Feature_Learning_Diagram.png&oldid=695252798.

[217] Omer N., Le Deley M.C., Piperno-Neumann S., Marec-Berard P., Italiano A., Corradini N., Bellera C., Brugières L., Gaspar N. (2017). Phase-II Trials in Osteosarcoma Recurrences: A Systematic Review of Past Experience. Eur. J. Cancer.

[218] Benjamin R.S. (2015). Osteosarcoma: Better Treatment through Better Trial Design. Lancet Oncol..

[219] Meyers P.A., Schwartz C.L., Krailo M.D., Healey J.H., Bernstein M.L., Betcher D., Ferguson W.S., Gebhardt M.C., Goorin A.M., Harris M. (2008). Children’s Oncology Group Osteosarcoma: The Addition of Muramyl Tripeptide to Chemotherapy Improves Overall Survival—AReport from the Children’s Oncology Group. J. Clin. Oncol..

[220] Gaspar N., Hawkins D.S., Dirksen U., Lewis I.J., Ferrari S., Le Deley M.C., Kovar H., Grimer R., Whelan J., Claude L. (2015). Ewing Sarcoma: Current Management and Future Approaches through Collaboration. J. Clin. Oncol..

[221] Womer R.B., West D.C., Krailo M.D., Dickman P.S., Pawel B.R., Grier H.E., Marcus K., Sailer S., Healey J.H., Dormans J.P. (2012). Children’s Oncology Group Randomized Controlled Trial of Interval-Compressed Chemotherapy for the Treatment of Localized Ewing Sarcoma: AReport from the Children’s Oncology Group. J. Clin. Oncol..

[222] Granowetter L., Womer R., Devidas M., Krailo M., Wang C., Bernstein M., Marina N., Leavey P., Gebhardt M., Healey J. (2009). Dose-Intensified Compared with Standard Chemotherapy for Nonmetastatic Ewing Sarcoma Family of Tumors: A Children’s Oncology Group Study. J. Clin. Oncol..

[223] Grier H.E., Krailo M.D., Tarbell N.J., Link M.P., Fryer C.J., Pritchard D.J., Gebhardt M.C., Dickman P.S., Perlman E.J., Meyers P.A. (2003). Addition of Ifosfamide and Etoposide to Standard Chemotherapy for Ewing’s Sarcoma and Primitive Neuroectodermal Tumor of Bone. N. Engl. J. Med..

[224] Casali P.G., Bielack S., Abecassis N., Aro H.T., Bauer S., Biagini R., Bonvalot S., Boukovinas I., Bovee J.V.M.G., Brennan B. (2018). Bone Sarcomas: ESMO-PaedCan-EURACAN Clinical Practice Guidelines for Diagnosis, Treatment and Follow-Up. Ann. Oncol..

[225] Paladugu P., Kumar R., Ong J., Waisberg E., Sporn K. (2025). Virtual Reality-Enhanced Rehabilitation for Improving Musculoskeletal Function and Recovery after Trauma. J. Orthop. Surg. Res..

[226] Cotterill S.J., Ahrens S., Paulussen M., Jürgens H.F., Voûte P.A., Gadner H., Craft A.W. (2000). Prognostic Factors in Ewing’s Sarcoma of Bone: Analysis of 975 Patients from the European Intergroup Cooperative Ewing’s Sarcoma Study Group. J. Clin. Oncol..

[227] Oberlin O., Deley M.C.L., Bui B.N., Gentet J.C., Philip T., Terrier P., Carrie C., Mechinaud F., Schmitt C., Babin-Boillettot A. (2001). French Society of Paediatric Oncology Prognostic Factors in Localized Ewing’s Tumours Peripheral Neuroectodermal Tumours: The Third Study of the French Society of Paediatric Oncology (EW88 Study). Br. J. Cancer.

[228] Rodriguez-Galindo C., Liu T., Krasin M.J., Wu J., Billups C.A., Daw N.C., Spunt S.L., Arndt C., Santana V.M., Navid F. (2007). Analysis of Prognostic Factors in Ewing Sarcoma Family of Tumors: Review of St. Jude Children’s Research Hospital Studies. Cancer.

[229] Averaged Chest Radiographs for all Participants and Heatmap of Regions’ Association with Aging (Saliency Maps from the External Test Dataset) [Internet]. Wikimedia Commons. 21 September 2024. https://commons.wikimedia.org/w/index.php?title=File:Averaged_chest_radiographs_for_all_participants_and_heatmap_of_regions%27_association_with_aging_(saliency_maps_from_the_external_test_dataset).jpg&oldid=926962397.

[230] Ladenstein R., Pötschger U., Le Deley M.C., Whelan J., Paulussen M., Oberlin O., van den Berg H., Dirksen U., Hjorth L., Michon J. (2010). Primary Disseminated Multifocal Ewing Sarcoma: Results of the Euro-EWING 99 Trial. J. Clin. Oncol..

[231] Fritz B., Yi P.H., Kijowski R., Fritz J. (2023). Radiomics and Deep Learning for Disease Detection in Musculoskeletal Radiology: An Overview of Novel MRI- and CT-Based Approaches. Investig. Radiol..

[232] Wang S., Sun M., Sun J., Wang Q., Wang G., Wang X., Meng X., Wang Z., Yu H. (2024). Advancing musculoskeletal tumor diagnosis: Automated segmentation and predictive classification using deep learning and radiomics. Comput. Biol. Med..

[233] Lemore A., Vogt N., Oster J., Germain E., Fauvel M., Gillet R., Sirveaux F., Marie B., Sans N., Faruch M. (2025). Enhanced CT and MRI Focal Bone Tumor Classification with Machine Learning–based Stratification: A Multicenter Retrospective Study. Radiology.

[234] Wang H., He Y., Wan L., Li C., Li Z., Li Z., Xu H., Tu C. (2025). Deep learning models in classifying primary bone tumors and bone infections based on radiographs. npj Precis. Oncol..

[235] von Schacky C.E., Wilhelm N.J., Schäfer V.S., Leonhardt Y., Gassert F.G., Foreman S.C., Gassert F.T., Jung M., Jungmann P.M., Russe M.F. (2021). Multitask Deep Learning for Segmentation and Classification of Primary Bone Tumors on Radiographs. Radiology.

[236] Leavey P.J., Mascarenhas L., Marina N., Chen Z., Krailo M., Miser J., Brown K., Tarbell N., Bernstein M.L., Granowetter L. (2008). Prognostic Factors for Patients with Ewing Sarcoma (EWS) at First Recurrence Following Multi-Modality Therapy: A Report from the Children’s Oncology Group. Pediatr. Blood Cancer.

[237] Barker L.M., Pendergrass T.W., Sanders J.E., Hawkins D.S. (2005). Survival after Recurrence of Ewing’s Sarcoma Family of Tumors. J. Clin. Oncol..

[238] Stahl M., Ranft A., Paulussen M., Bölling T., Vieth V., Bielack S., Görtitz I., Braun-Munzinger G., Hardes J., Jürgens H. (2011). Risk of Recurrence and Survival after Relapse in Patients with Ewing Sarcoma. Pediatr. Blood Cancer.

[239] Ferrari S., Luksch R., Sundby Hall K., Fagioli F., Prete A., Tamburini A., Tienghi A., Erba P., Melchionda F., Di Giulio G. (2015). Post-Relapse Survival in Patients with Ewing Sarcoma. Pediatr. Blood Cancer.

[240] Bacci G., Ferrari S., Longhi A., Donati D., De Paolis M., Forni C., Versari M., Setola E., Briccoli A., Barbieri E. (2003). Therapy and Survival after Recurrence of Ewing’s Tumors: The Rizzoli Experience in 195 Patients Treated with Adjuvant and Neoadjuvant Chemotherapy from 1979 to 1997. Ann. Oncol..

[241] McTiernan A., Driver D., Michelagnoli M.P., Kilby A.M., Whelan J.S. (2006). High Dose Chemotherapy with Bone Marrow or Peripheral Stem Cell Rescue Is an Effective Treatment Option for Patients with Relapsed or Progressive Ewing’s Sarcoma Family of Tumours. Ann. Oncol..

[242] Gardner S.L., Carreras J., Bjerre J., Cheung N.K., Dunkel I.J., Finlay J., Gardner S., Goldman S., Grant G., Kalapurakal J. (2010). Phase II Study of Oral Capecitabine in Children with Relapsed/Refractory Ewing Sarcoma or Osteosarcoma: A Report from the Children’s Oncology Group (COG). J. Clin. Oncol..

[243] Fox E., Patel S., Wathen J.K., Schuetze S., Chawla S., Harmon D., Reinke D., Chugh R., Benjamin R.S., Helman L.J. (2012). Phase II Study of Sequential Gemcitabine Followed by Docetaxel for Recurrent Ewing Sarcoma, Osteosarcoma, or Unresectable or Locally Recurrent Chondrosarcoma: Results of Sarcoma Alliance for Research Through Collaboration Study 003. Oncologist.

[244] Casey D.A., Wexler L.H., Merchant M.S., Chou A.J., Merola P.R., Price A.P., Meyers P.A. (2009). Irinotecan and Temozolomide for Ewing Sarcoma: The Memorial Sloan-Kettering Experience. Pediatr. Blood Cancer.

[245] Schafer E.S., Rau R.E., Berg S., Liu X., Minard C.G., D’Adamo D., Scott R., Reyderman L., Martinez G., Devarajan S. (2018). A Phase 1 Study of Eribulin Mesylate (E7389), a Novel Microtubule-Targeting Chemotherapeutic Agent, in Children with Refractory or Recurrent Solid Tumors: A Children’s Oncology Group Phase 1 Consortium Study (ADVL1314). Pediatr. Blood Cancer.

[246] Grignani G., Palmerini E., Dileo P., Asaftei S.D., D’Ambrosio L., Pignochino Y., Mercuri M., Picci P., Fagioli F., Casali P.G. (2012). A Phase II Trial of Sorafenib in Relapsed and Unresectable High-Grade Osteosarcoma after Failure of Standard Multimodal Therapy: An Italian Sarcoma Group Study. Ann. Oncol..

[247] Duffaud F., Mir O., Boudou-Rouquette P., Piperno-Neumann S., Penel N., Bompas E., Delcambre C., Kalbacher E., Italiano A., Collard O. (2019). Efficacy and Safety of Regorafenib in Adult Patients with Metastatic Osteosarcoma: A Non-Comparative, Randomised, Double-Blind, Placebo-Controlled, Phase 2 Study. Lancet Oncol..

[248] Davis L.E., Bolejack V., Ryan C.W., Ganjoo K.N., Loggers E.T., Chawla S., Agulnik M., Livingston M.B., Park S., Reed D.R. (2019). Randomized Double-Blind Phase II Study of Regorafenib in Patients with Metastatic Osteosarcoma. J. Clin. Oncol..

[249] Wedekind M.F., Wagner L.M., Cripe T.P. (2018). Immunotherapy for Osteosarcoma: Where Do We Go from Here?. Pediatr. Blood Cancer.

[250] Ahmed N., Brawley V.S., Hegde M., Robertson C., Ghazi A., Gerken C., Liu E., Dakhova O., Ashoori A., Corder A. (2015). Human Epidermal Growth Factor Receptor 2 (HER2)–Specific Chimeric Antigen Receptor–Modified T Cells for the Immunotherapy of HER2-Positive Sarcoma. J. Clin. Oncol..

[251] Merchant M.S., Wright M., Baird K., Wexler L.H., Rodriguez-Galindo C., Bernstein D., Delbrook C., Lodish M., Bishop R., Wolchok J.D. (2016). Phase I Clinical Trial of Ipilimumab in Pediatric Patients with Advanced Solid Tumors. Clin. Cancer Res..

[252] Tawbi H.A., Burgess M., Bolejack V., Van Tine B.A., Schuetze S.M., Hu J., D’Angelo S., Attia S., Riedel R.F., Priebat D.A. (2017). Pembrolizumab in Advanced Soft-Tissue Sarcoma and Bone Sarcoma (SARC028): A Multicentre, Two-Cohort, Single-Arm, Open-Label, Phase 2 Trial. Lancet Oncol..

[253] D’Angelo S.P., Mahoney M.R., Van Tine B.A., Atkins J., Milhem M.M., Jahagirdar B.N., Antonescu C.R., Horvath L.E., Tap W.D., Schwartz G.K. (2018). Nivolumab with or without Ipilimumab Treatment for Metastatic Sarcoma (Alliance A091401): Two Open-Label, Non-Comparative, Randomised, Phase 2 Trials. Lancet Oncol..

[254] Le Cesne A., Marec-Berard P., Blay J.Y., Gaspar N., Bertucci F., Penel N., Bompas E., Cousin S., Toulmonde M., Bessede A. (2018). Programmed Cell Death 1 (PD-1) Targeting in Patients with Advanced Osteosarcomas: Results from the PEMBROSARC Study. J. Clin. Oncol..

[255] Boye K., Longhi A., Guren T., Lorenz S., Næss S., Pierini M., Taksdal I., Lobmaier I., Cesari M., Paioli A. (2021). Pembrolizumab in Advanced Osteosarcoma: Results of a Single-Arm, Open-Label, Phase 2 Trial. Cancer Immunol. Immunother..

[256] Thanindratarn P., Dean D.C., Nelson S.D., Hornicek F.J., Duan Z. (2019). Advances in Immune Checkpoint Inhibitors for Bone Sarcoma Therapy. J. Bone Oncol..

[257] Zhou M., Bui N., Bolourchi S., Lohman M., Elicer O., Dupuy A.G., Lizardo M.M., Allen J., Caldwell A., Withers S. (2023). Targeting the Tumor Microenvironment of Ewing Sarcoma with the Combination of Dinutuximab and Trabectedin: A New Immunotherapeutic Strategy. J. Clin. Oncol..

[258] Rainusso N., Brawley V.S., Ghazi A., Hicks J., Gottschalk S., Rosen J.M., Ahmed N., Hegde M., Wu M.F., Liu H. (2012). Immunotherapy Targeting HER2 with Genetically Modified T Cells Eliminates Tumor-Initiating Cells in Osteosarcoma. Cancer Gene Ther..

[259] Long A.H., Highfill S.L., Cui Y., Smith J.P., Walker A.J., Ramakrishna S., El-Etriby R., Orentas R.J., Tran T., Mackall C.L. (2016). Reduction of MDSCs with All-Trans Retinoic Acid Improves CAR Therapy Efficacy Against Sarcomas. J. Clin. Oncol..

[260] Majzner R.G., Theruvath J.L., Nellan A., Heitzeneder S., Cui Y., Mount C.W., Rietberg S.P., Linde M.H., Xu P., Rota C. (2022). GD2-CAR T Cell Therapy for H3K27M-Mutated Diffuse Midline Gliomas. Nature.

[261] Hegde M., Joseph S.K., Pashankar F., DeRenzo C., Sanber K., Navai S., Byrd T.T., Hicks J., Xu M.L., Gerken C. (2020). Tumor Response and Endogenous Immune Reactivity Following Administration of HER2 CAR T Cells in a Child with Metastatic Rhabdomyosarcoma. Nat. Commun..

[262] Navai S.A., Derenzo C., Joseph S., Sanber K., Byrd T., Zhang H., Mata M., Gerken C., Shree A., Mathew P.R. (2018). Administration of HER2-Chimeric Antigen Receptor (CAR) Expressing T Cells for Sarcoma Is Safe, with Preliminary Evidence of Efficacy in HER2 Low-Expressing Tumors. J. Clin. Oncol..

[263] Chulanetra M., Morchang A., Sayour E., Eldjerou L., Milner R., Lagmay J., Slayton W., Ligon J., Simon E., Schafer B. (2020). GD2 Chimeric Antigen Receptor T-Cell Therapy for Tyrosine Hydroxylase-Positive Stage 4 Neuroblastomas and Sarcomas. J. Clin. Oncol..

[264] Hingorani P., Janeway K., Crompton B.D., Kadoch C., Mackall C.L., Khan J., Shern J.F., Schiffman J., Mirabello L., Savage S.A. (2016). Current State of Pediatric Sarcoma Biology and Opportunities for Future Discovery: A Report from the Sarcoma Translational Research Workshop. Cancer Genet..

[265] Sporn K., Kumar R., Paladugu P., Ong J., Sekhar T., Vaja S., Hage T., Waisberg E., Gowda C., Jagadeesan R. (2025). Artificial Intelligence in Orthopedic Medical Education: A Comprehensive Review of Emerging Technologies and Their Applications. Int. Med. Educ..

[266] Roberts R.D., Lizardo M.M., Reed D.R., Hingorani P., Glover J., Allen-Rhoades W., Fan T., Khanna C., Sweet-Cordero E.A., Cash T. (2019). Provocative Questions in Osteosarcoma Research: An Update from the Children’s Oncology Group. Cancer.

